# ER-Targeted Beclin 1 Supports Autophagosome Biogenesis in the Absence of ULK1 and ULK2 Kinases

**DOI:** 10.3390/cells8050475

**Published:** 2019-05-17

**Authors:** Tahira Anwar, Xiaonan Liu, Taina Suntio, Annika Marjamäki, Joanna Biazik, Edmond Y. W. Chan, Markku Varjosalo, Eeva-Liisa Eskelinen

**Affiliations:** 1 Molecular and Integrative Biosciences Research Programme, University of Helsinki, 00014 Helsinki, Finland; tahira.anwar@helsinki.fi (T.A.); marjamaki.annika@gmail.com (A.M.); jmbiazik@gmail.com (J.B.); 2 Institute of Biotechnology & HiLIFE, University of Helsinki, 00014 Helsinki, Finland; xiaonan.liu@helsinki.fi (X.L.); markku.varjosalo@helsinki.fi (M.V.); 3 Institute of Biotechnology, University of Helsinki, 00014 Helsinki, Finland; taina.suntio@helsinki.fi; 4 Department of Biomedical and Molecular Sciences and Department of Pathology and Molecular Medicine, Queen’s University, Kingston, ON K7L 3N6, Canada; eywc@queensu.ca; 5 Strathclyde Institute of Pharmacy and Biomedical Sciences, University of Strathclyde, Glasgow G4 0RE, UK; 6 Institute of Biomedicine, University of Turku, 20520 Turku, Finland

**Keywords:** autophagy, Beclin 1, ULK1, ULK2, endoplasmic reticulum, mitochondria

## Abstract

Autophagy transports cytoplasmic material and organelles to lysosomes for degradation and recycling. Beclin 1 forms a complex with several other autophagy proteins and functions in the initiation phase of autophagy, but the exact role of Beclin 1 subcellular localization in autophagy initiation is still unclear. In order to elucidate the role of Beclin 1 localization in autophagosome biogenesis, we generated constructs that target Beclin 1 to the endoplasmic reticulum (ER) or mitochondria. Our results confirmed the proper organelle-specific targeting of the engineered Beclin 1 constructs, and the proper formation of autophagy-regulatory Beclin 1 complexes. The ULK kinases are required for autophagy initiation upstream of Beclin 1, and autophagosome biogenesis is severely impaired in ULK1/ULK2 double knockout cells. We tested whether Beclin 1 targeting facilitated its ability to rescue autophagosome formation in ULK1/ULK2 double knockout cells. ER-targeted Beclin 1 was most effective in the rescue experiments, while mitochondria-targeted and non-targeted Beclin 1 also showed an ability to rescue, but with lower activity. However, none of the constructs was able to increase autophagic flux in the knockout cells. We also showed that wild type Beclin 1 was enriched on the ER during autophagy induction, and that ULK1/ULK2 facilitated the ER-enrichment of Beclin 1 under basal conditions. The results suggest that one of the functions of ULK kinases may be to enhance Beclin 1 recruitment to the ER to drive autophagosome formation.

## 1. Introduction

Macroautophagy, here referred to as autophagy, is a major pathway used by cells to degrade large cytoplasmic cargoes in lysosomes. Autophagy is upregulated during stress, and it also serves an important quality control mechanism since it eliminates unwanted or damaged organelles, protein aggregates and other unnecessary elements. Defects in autophagy have been implicated in several diseases including cancer, neurodegeneration and muscle disorders [1,2]. Upon induction of autophagy, a flat membrane cistern called the phagophore grows around the cytoplasmic cargo, forming a closed, double-membraned autophagosome. The completed autophagosome then fuses with a lysosome forming an autolysosome, where the cytoplasmic cargo is degraded. This releases macromolecules back to the cytoplasm, and the cell can use them for biosynthesis and energy production [3].

Autophagy initiation is tightly regulated by several protein complexes including the ULK1 complex (unc-51-like autophagy activating kinase 1) and the class III phosphoinositide (PI) 3-kinase complex I. The ULK1 complex acts upstream of the PI3-kinase complex I. ULK1 is a serine/threonine kinase, and mammals also possess ULK2 which is less characterized. ULK1 is found in a complex with FAK family-interacting protein of 200 kDa (FIP200), ATG101 and ATG13. In nutrient-rich conditions, ULK1 is inhibited by the mechanistic target of rapamycin complex 1 (mTORC1). When the cellular nutrients are depleted, mTORC1 becomes inactive, while ULK1 is activated and starts a cascade of autophosphorylations within the complex itself. Activated ULK1 also phosphorylates several downstream effectors including Beclin 1, as well as two Beclin 1 binding partners, VPS34 and ATG14 [4].

Beclin 1, the mammalian orthologue of yeast Atg6/Vps30, is the core component of the class III PI3-kinase complex I [5]. It is encoded by a haploinsufficient tumor suppressor gene that is mono-allelically deleted in various cancers [6,7]. Beclin 1 contains a nuclear export signal that is required for its autophagy function [8]. The importance of Beclin 1 in autophagy resides in its interaction with the class III PI3-kinase VPS34 (vacuolar sorting protein 34, also known as PI3KC3) [9,10]. VPS34 produces phosphatidylinositol 3-phosphate (PI3P) by phosphorylating the three-position hydroxyl group of the inositol ring in phosphatidylinositol. PI3P is required for the recruitment of effectors involved in autophagosome biogenesis. Inhibition of the VPS34 kinase activity severely hinders autophagosome formation [11]. Class III PI3-kinase complex I also contains the scaffold protein VPS15 (also known as PI3R4), which is essential for the assembly of the complex as well as for the activity of the VPS34 kinase [12]. The class III PI3-kinase complex I also contains ATG14, also known as Beclin 1-associated autophagy-related key regulator (Barkor) [5,13].

Several proteins of the Beclin 1 complex are targets of ULK1 phosphorylation. Upon starvation, ULK1 phosphorylates Beclin 1 on Serine 14 (Serine 15 in human Beclin 1), thus enhancing the activity of VPS34 and promoting autophagy [14]. ULK1 also phosphorylates VPS34 on Serine 249 but the significance of this modification is not clear [15]. Furthermore, ULK1 also robustly phosphorylates ATG14 on Serine 29 [16]. This allows the activation of the class III PI3-kinase complex I and subsequent autophagosome initiation. Autophagy induction is inhibited upon amino acid starvation in ULK1 and ULK2 double knockout mouse embryonic fibroblasts (MEF-ULK1/2-KO) [17]. Also, production of PI3P is reduced in the double knockout cells.

The origin of phagophore membranes is not fully understood and several organelles including endoplasmic reticulum (ER), ER exit sites, ER-Golgi intermediate compartment, mitochondria, endosomes and the plasma membrane have been implicated to play a role in autophagosome biogenesis. Many studies point to the ER as the most likely site for autophagosome formation [18,19,20]. Phagophores emerge from a PI3P-enriched ER subdomain named the omegasome; this domain is positive for DFCP1 (also called ZFYVE1), an ER-resident protein that binds to PI3P [21]. The omegasome functions as a cradle for the forming phagophore [22,23]. Autophagy protein complexes are recruited sequentially to the omegasome during phagophore biogenesis. Upon starvation, the ULK1 complex is among the first autophagy complexes to closely associate with the omegasome [24]. Recruitment of the ULK1 complex is followed by the class 3 PI3-kinase complex I [25,26,27]. Targeting of the PI3-kinase complex I to the ER is mediated by ATG14 [28], and possibly also by lipid binding of Beclin 1 [29]. In addition to the ER, mitochondria have also been suggested to play a role in autophagosome biogenesis. Mitochondria have been proposed to donate membrane for forming phagophores [30], and autophagosomes have been suggested to form in ER-mitochondria contact sites [31].

We aimed to elucidate the role of the subcellular localization of the class 3 PI3-kinase complex I in autophagosome biogenesis. To study this, we constructed Beclin 1 chimeras by adding peptide sequences to the C-terminal end of Beclin 1, which target the chimera to the ER or mitochondria. ER targeting was chosen since the role of the ER and omegasomes in phagophore biogenesis is well established. Mitochondrial targeting was used for comparison. We also used the Beclin 1 chimeras to study whether forced targeting of Beclin 1 to the ER is able to rescue autophagosome formation in the absence of ULK1 and ULK2. This was done using MEF-ULK1/2-KO cells [17]. Our results showed that all our Beclin 1 constructs bind to known autophagy-related Beclin 1 interactors. More importantly, Beclin 1 targeted to the ER was able to partially rescue autophagosome formation in ULK1 and ULK2 double knockout cells. We also observed that during starvation, Beclin 1 is enriched in the ER, and that ULK1/2 facilitate the ER enrichment of Beclin 1.

## 2. Materials and Methods

### 2.1. Cell Culture, Stable Cell Lines, Drugs and Autophagy Induction

Wild-type and ULK1/2-KO MEF [17] and human embryonic kidney 293 (HEK293 ) cells, parental cell line and lines stably and inducibly expressing Twin-StrepII-HA-tagged Beclin 1 constructs, were grown in Dulbecco´s modified Eagle´s medium (DMEM, Sigma, St. Louis, MO, USA, D6546) with 10% fetal bovine serum (FBS, Sigma F4524), penicillin/streptomycin solution (Sigma, P0781) and l-glutamine (Sigma, 67513). Cells were kept in an incubator with 5% CO_2_ at +37 °C. HEK293 cells stably expressing Twin-StrepII-HA-tagged Beclin 1 constructs were induced with 2 μg/mL tetracycline for 24 h prior to experiments. Autophagy was induced by starvation in serum and amino acid free Earle´s balanced salt solution (EBSS, Gibco, Grand Island, NY, USA, 2401043). Bafilomycin A1 (100 nM, 1 to 2 h depending on the experimental setting) was used to inhibit lysosomal proton pump (Enzo Biochem, Farmingdale, NY, USA, BML-CM110-0100). Carbonyl cyanide 3-chlorophenylhydrazone (CCCP, 15 μM or 30 μM, 30 min to 7 h depending on the experimental setting) was used to inhibit oxidative phosphorylation in mitochondria (Acros Organics, Geel, Belgium, AC228131000). Tetramethylrhodamine ethyl ester (TMRE, 50 nM, 30 min) was used to monitor mitochondrial membrane potential (Abcam, Cambridge, UK, 113852). Tunicamycin (10 μg/mL, 6 h) was used to induce ER stress (Assay Designs, Ann Arbor, MI, USA, 908-297).

### 2.2. Plasmid Construction

The peptide sequence used for Beclin 1 targeting to the ER (Beclin 1-ER) was ITTIDSSSSWWTNWVIPAISAVAVALMYRLYMAED from Cytochrome b5 [32]. Sequence for mitochondrial targeting (Beclin 1-MITO) was LILAMLAIGVFSLGAFIKIIQLRKNN from the Listerial protein ActA that is involved in the polymerization of the actin cytoskeleton in the host cells of the bacteria. ActA protein lacking the internal proline repeat sequences localizes to the outer mitochondrial membrane [33]. These peptide sequences have previously been used to target Bcl2 to ER and mitochondria [34]. The targeted Beclin 1 constructs were created with polymerase chain reaction (PCR) using specific overlapping oligonucleotide primers, and cloned into pCDNA3.1(+) FLAG-Beclin 1 vector to be placed in the C-terminus of Beclin 1. The FLAG-Beclin 1 vector was a gift from Harold Jefferies, The Francis Crick Institute, London, UK. These Beclin 1 constructs were then cloned by PCR amplification into the pEGFP-C1 vector (BD Biosciences Clontech, Palo Alto, CA, USA). A small linker was inserted between eGFP and Beclin 1 sequences to allow proper folding of both ends of the eGFP-Beclin 1 fusion proteins. The eGFP- Beclin 1 constructs contained the eGFP tag at the N-terminus, followed by the linker and the Beclin 1 sequence. The organelle targeting sequence was at the C-terminus of Beclin 1. The eGFP-tagged Beclin 1 constructs had no FLAG tag.

The targeted control constructs encoding the ER targeting peptide from Cytochrome b5, or the mitochondrial targeting sequence from the Listerial protein ActA, but without Beclin 1 sequence, were amplified by PCR with overlapping oligonucleotide primers and cloned into pEGFP-C1 vector. Thus, the control constructs had eGFP in the N-terminus of the targeting peptide. All primers were purchased from TAG Copenhagen, Denmark, and all constructs were confirmed by sequencing (GATC Biotech, Cologne, Germany). Refer to Table S1 of Supplemental Materials for a list of primers used in the plasmid construction.

### 2.3. Transient and Stable Transfection

eGFP-Beclin 1 constructs were transfected into MEF cells with Xfect^TM^ transfection reagent (BD Biosciences Clontech 631318) according to the manufacturer’s instructions, 24 h after seeding the cells, and 24 h before the experimental treatments.

In order to create HEK293 cell lines stably and inducibly expressing the Beclin 1 constructs, we created plasmids encoding Beclin 1 tagged with Twin-StrepII and HA. The cDNA encoding non-targeted Beclin 1 (Beclin 1-WT), Beclin 1-ER or Beclin 1-MITO was cloned into the Gateway entry vector pDONR 221 with a two-step PCR reaction (Invitrogen, Whaltham, MA, USA, 11789-020). Beclin 1 sequences were first amplified with specific oligonucleotide primers (TAG Copenhagen, Copenhagen, Denmark). These amplified fragments were then used for the BP clonase reaction, in order to insert them into the entry clone pDONR 221. LR clonase recombination was performed between the entry clones and the in-house–designed destination vector. The destination vector containing the affinity tags Twin-StrepII and HA was used for all the constructs. All primers were purchased from TAG Copenhagen, and all constructs were confirmed by sequencing (GATC Biotech). Refer to Table S1 of Supplemental Materials for a list of the primers used in the Gateway cloning. For affinity purification of the Beclin 1 constructs, tetracycline-inducible Flp-In™ T-REx 293 cell lines were generated as described previously [35].

### 2.4. Immunofluorescence and Imaging

Wild-type and ULK1/2-KO MEFs were plated on glass coverslips and HEK293 cells stably and inducibly expressing Twin-StrepII-HA-tagged Beclin 1 constructs were plated on Poly-l-lysine (Sigma, P4707) coated glass coverslips. 24 h after transfection (MEF) or induction with tetracycline (HEK293), cells were starved in EBSS for 1 h, or kept in full culture medium, and then fixed in 4% paraformaldehyde (PFA) in phosphate-buffered saline (PBS; 140 mM NaCl, 2.7 mM KCl, 4.6 mM Na_2_HPO_4_ 2H_2_O, 1.5 mM KH_2_PO_4_) at room temperature for 30 min, or in methanol at −20 °C for 5 min. Cells fixed in PFA were permeabilized with 0.2% saponin (Amresco, Solon, OH, USA, 0163) in PBS for 10 min and blocked with 3% bovine serum albumin (BSA, Biowest, Nuaillé, France, P6154) in PBS/saponin for 30 min. Cells were labelled with rabbit anti-TOM20 (1:2000, Santa Cruz Biotechnology, Dallas, TX, USA, FL-145), mouse anti-LC3 (1:100, Cosmo Bio, Tokyo, Japan, CTB-LC3-2-IC), or mouse anti-HA (1:500, Biolegend, San Diego, CA, USA, MMS-101R). Cells fixed in PFA were also permeabilized with 0.1% Triton X-100 (MP Biomedicals, Solon, OH, USA, 194854) for 5 min, blocked in 3% BSA in PBS for 30 min and labelled with rabbit anti-BAP31 (1:400, a generous gift of Esa Kuismanen, [36]), rabbit anti-calreticulin (1:100, Invitrogen, PA3-900), mouse anti-PDI clone 1D3 (1:20, a generous gift from Stephen Fuller, University of Oxford, UK), mouse anti-CHOP (1:2000, Cell Signaling Technology, Leiden, Netherlands, L63F7) or mouse anti-GM130 (1:100, BD Transduction Laboratories, San Jose, CA, USA, 610822). Primary antibody incubation was followed by incubation with secondary antibodies conjugated to Alexa Fluor 594 (goat anti mouse AF-594, Invitrogen, A-11032, and goat anti rabbit AF-594, Invitrogen, A-11037) or Alexa Fluor 488 (goat anti mouse AF-488, Invitrogen, A-11029). Coverslips were mounted on microscope slides with Mowiol (Calbiochem, San Diego, CA, USA, 475904) containing the antifading agent 1,4-Diazabicyclo [2.2.2] octane (DABCO, Sigma, D-2522) and the nuclear stain 4ʼ,6-diamidine-2-phenyl indole (DAPI, Pierce, Whaltham, MA, USA, 62247).

Images were taken with a confocal microscope (Leica TCS SP5, Leica Microsystems, Wetzlar, Germany, HCX APO 63x/1,30 Corr CS 21 glycerol objective or Leica TCS SP5 II HCS-A, HCX PL APO 63x/1,2 W Corr/0,17 CS (water) Lbd.bl.). Images for the LC3 CellProfiler quantification were taken with upright fluorescence wide field microscope (Leica DM6000B, 63x/1.40-0.60 HCX PL APO Lbd.bl. Oil objective wd = 0.10). Images for the TMRE CellProfiler quantification and for the colocalization analysis were taken with a confocal microscope (Leica TCS SP5 II HCS-A, HCX PL APO 63x/1,2 W Corr/0,17 CS (water) Lbd.bl.).

### 2.5. Immunofluorescence Image Quantification

LC3 labelling in cells expressing the eGFP-Beclin 1 constructs was quantified with CellProfiler software. Data was collected by imaging 50 cells per sample. A pipeline was built in CellProfiler for image analysis [37,38]. DAPI (nuclei) images were first smoothed with a Gaussian filter. Nuclei were identified with IdentifyPrimaryObjects module using default automatic threshold settings, and cell borders were identified with IdentifySecondaryObjects module with adaptive thresholding. To detect LC3 spots (Alexa Fluor 594) in the images, we ran an ImageJ plugin with Otsu thresholding [39], followed by IdentifyPrimaryObjects module for LC3 spots with a manually set correction factor. eGFP expressing cells were recognized with MeasureObjectIntensity module and filtered by mean intensity of the eGFP image. The results were shown as proportion of LC3 in vesicles. The values for vesicular LC3 staining, (Sum_Intensity_IntegratedIntensity_LC3) and diffuse cytoplasmic LC3 staining [Intensity_IntegratedIntensity_LC3 (cellsGFPminusSpots)] were obtained from the CellProfiler analysis. These were then summed together in order to obtain the total amount of LC3 staining within the cell of interest. Finally, intensity of vesicular LC3 staining was divided by the total amount of LC3, in order to obtain the proportion of vesicular LC3 staining for the cell of interest. This protocol was followed for both cells expressing eGFP-tagged constructs and non-expressing cells on the same coverslips.

TMRE intensity (Intensity_IntegratedIntensity_red) per cell was estimated using CellProfiler, in wild type MEF expressing the eGFP-Beclin 1 constructs. Confocal optical sections were taken from the cells using a constant gain (voltage) for the TMRE channel and the same zoom for all images. Twenty live cells were imaged per sample. A pipeline was built in CellProfiler for image analysis. Cell borders for expressing cells, non-expressing cells, and CCCP-treated cells were identified manually with IdentifyObjectsManually module. For the CCCP images, ImageMath module was used to enhance the image signal in order to identify cell borders manually.

Wild type Beclin 1 colocalization with the ER markers was assessed with Imaris 9.2 software (Bitplane, Belfast, UK). Confocal images were taken using a constant zoom and width-height format. On the Imaris 9.2 software, 20% of the highest value for each channel was used as threshold for the quantification. Colocalization was quantified as Pearson’s correlation coefficient.

### 2.6. Correlative Light Electron Microscopy (CLEM)

Wild-type and ULK1/2-KO MEF cells were seeded on gridded glass-bottom dishes (MatTek Corporation, Ashland, MA, USA). The cells were transfected with the eGFP-tagged Beclin 1 constructs; 24 h after transfection, cells were starved for 1 h and fixed in 2% paraformaldehyde and 1.5% glutaraldehyde in 0.1 M sodium cacodylate buffer, pH 7.4. After fixation, fluorescence images were taken with a spinning disk confocal microscope (3I Marianas, Denver, CO, USA, 63X/1.2 W C-Apochromat Corr WD = 0.28 M27), and then the cells were postfixed in 1% osmium tetroxide in cacodylate buffer at room temperature for 1 h. Cells were washed in cacodylate buffer, dehydrated in a graded series of ethanol, and washed once with acetone. Beam capsules filled with resin were placed upside down onto the region of interest. The dishes were incubated at room temperature for 2 h, and polymerized at +60 °C for 18 h. The polymerized capsules were detached from the Mattek dishes, and 70–90 nm sections were cut with a diamond knife, using the grid as guide in order to catch the cells of interest in the thin sections. The sections were stained with uranyl acetate and lead citrate. Images were taken on a JEM-1400 (Jeol, Tokyo, Japan) transmission electron microscope.

### 2.7. Western Blotting

Cells were scraped off the culture plate and pelleted by centrifugation. The pellets were lysed in 50 mM Tris-HCl, 150 mM NaCl, 1 mM EDTA, 1% Triton X-100, pH7.4, Complete EDTA-free protease inhibitor cocktail (Roche, Basel, Switzerland, 04693132001), on ice for 40 min. Lysates were centrifuged at 13,000 rpm at 4 °C for 15 min, and protein concentration was measured with Pierce^TM^ BCA protein assay kit (Thermo Scientific, Waltham, MA, USA, 23228). Laemmli buffer was added to the lysates (0.2 M Tris-HCl, pH 6.8, 8% sodium dodecyl sulfate (SDS), 2% mercaptoethanol, 0.02% bromophenol blue) and the samples were boiled at +95 °C for 5 min. Samples were resolved in sodium dodecyl sulfate polyacrylamide gel electrophoresis (SDS-PAGE) gels and transferred onto PVDF membranes (Thermo Scientific 88518). Membranes were blocked in 5% nonfat milk powder in Tris-buffered saline (100 mM Tris-HCl, 1.5 M NaCl, pH 7.6) containing 0.05% Tween 20 (Sigma Aldrich, St. Louis, MO, USA, P1379). Membranes were labelled with mouse anti-HA (1:5000, Biolegend, MMS-101R), rabbit anti-LC3 (1:1000, Sigma, L75432), rabbit anti-p62 (1:1000, Cell Signaling Technology, 5114), mouse anti-pan 14-3-3 (1:2000, Santa Cruz Biotechnology, B-8, sc-133233), mouse anti-β-tubulin clone E7 (1:1000, Developmental Studies Hybridoma Bank, University of Iowa, USA), mouse anti-OPA1 (1:1000, BD Transduction Laboratories, 612606) or rabbit anti-β-actin (1:500, ThermoFisher Scientific, Whaltham, MA, USA, PA5-16194). Goat anti-mousehorseradish peroxidase (HRP) and goat anti-rabbit-HRP conjugates were purchased from Jackson Immuno Research Laboratories (West Grove, PA, USA, 115-035-003, 111-035-003). Bands were detected with Immobilon Western HRP substrate kit (Millipore, Darmstadt, Germany, WBKLS0500).

### 2.8. Affinity Purification Mass Spectrometry (AP–MS)

24 h before harvesting, each stable cell line was seeded to 80% confluency on a total of 30 150-mm cell culture plates. Half of the plates were amino-acid starved, and the other half was left non-starved. Cells from five 150-mm confluent plates (~2 × 10^7^ cells) were pelleted as one biological sample. Thus, each bait protein had three biological replicates in two different conditions. Samples were snap frozen and stored at −80 °C. Affinity purification (AP) of Twin-StrepII-HA-tagged Beclin 1 constructs, and liquid chromatography–mass spectrometry (LC–MS), were performed as explained previously [40]. For protein identification, Thermo .RAW files were uploaded into Proteome Discoverer 1.4 (Thermo Scientific) and searched against the selected human part of UniProtKB/SwissProt database (http://www.uniprot.org/, version 2015-01) using SEQUEST. The following search parameters were included: trypsin was selected as the enzyme and a maximum of two miss cleavages were permitted, precursor mass tolerance at ±15 ppm and fragment mass tolerance at 0.05 Da. Carbamidomethylation of cysteine was defined as static modifications and oxidation of methionine was specified as variable modification. All reported data were based on high confidence peptides assigned in Proteome Discoverer with a 0.05% false discovery rate (FDR) by Percolator.

Significance Analysis of INTeractome (SAINT)-express version 3.6 [41] was used as statistical tools for identification of high-confidence interacting proteins (HCIPs) from our AP–MS data. 26 eGFP runs (13 N-terminal Twin-StrepII-HA-eGFP and 13 C-terminal eGFP-Twin-StrepII-HA runs) were used as control, and the final results are presented as table (Supplementary Table S2). Only proteins with Bayesian FDR < 0.05 were considered as HCIPs.

### 2.9. Statistical Analysis

The statistical analysis was done using one-way analysis of variance (ANOVA) for multiple comparisons between two or three groups, or two-tailed unpaired t-test. When three groups were compared, ANOVA was followed by Tukey Kramer post hoc test. Statistical significance was set at *p* < 0.05.

## 3. Results

### 3.1. Beclin 1 Constructs Targeted to the Endoplasmic Reticulum and Mitochondria Localize to Their Expected Subcellular Compartments

**Figure 1:**
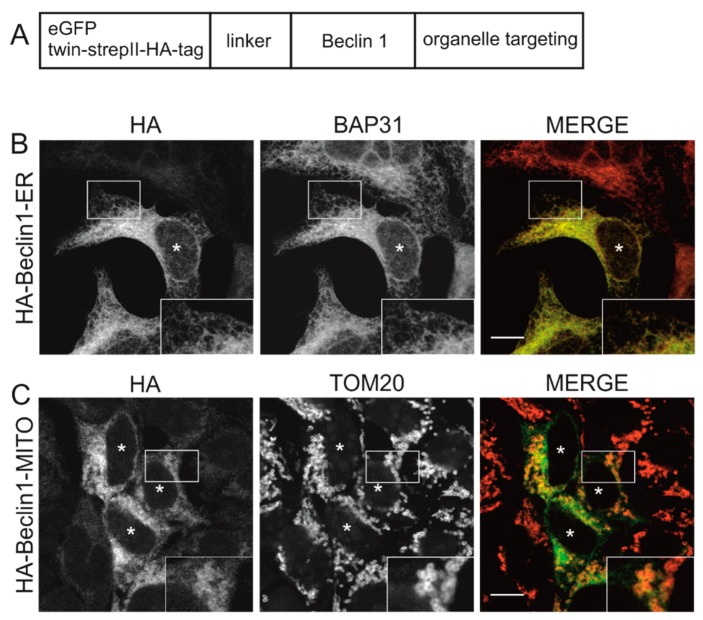
Subcellular localization of Beclin 1 targeted to endoplasmic reticulum and Beclin 1 targeted to mitochondria in HEK293 cells stably expressing the Twin-StrepII-HA-tagged Beclin 1 constructs. (**A**) Schematic representation of N-terminally epitope-tagged Beclin 1 constructs with C-terminal targeting peptides. (**B**,**C**) HEK293 cells stably expressing Twin-StrepII-HA-tagged Beclin 1-ER (endoplasmic reticulum) (**B**) or Beclin 1-MITO (**C**) were induced with tetracycline for 24 h. Cells were labelled with anti-HA, anti-BAP31 (ER marker), or anti-TOM20 (mitochondrial marker) as indicated. Images were taken with a confocal microscope and one optical section is shown. Cells expressing the Beclin 1 constructs are indicated by asterisks. Scale bars, 10 μm.

In order to study whether forced targeting of Beclin 1 to ER or mitochondria affect autophagy, we generated constructs of N-terminally epitope-tagged Beclin 1 with C-terminal targeting peptides (Figure 1A). ER and mitochondrial targeting peptides were from cytochrome b5 and Listerial protein ActA, respectively, as described in Material and Methods. Stable and inducible HEK293 cells lines were created using Twin-StrepII-HA double-tagged Beclin 1. The expression was induced with tetracycline for 24 h, and the localization of the construct was then studied by immunofluorescence using anti-HA. We first studied the subcellular localization of wild-type Beclin 1 (no targeting peptide) in HEK293 cells. The wild-type Beclin 1 construct (Twin-StrepII-HA-Beclin 1-WT) displayed a predominantly diffuse cytoplasmic localization (Figure S1A–C). Double immunofluorescence staining revealed limited colocalization with ER markers BAP31 and calreticulin, and no colocalization with the outer mitochondrial membrane protein TOM20 (Figure S1A–C). ER-targeted Beclin 1 (Twin-StrepII-HA-Beclin 1-ER) colocalized well with the ER protein BAP31 as expected (Figure 1B). Mitochondrial-targeted Twin-StrepII-HA-Beclin 1-MITO substantially colocalized with TOM20 as expected (Figure 1C). Stable expression of Twin-StrepII-HA-Beclin 1-ER or Twin-StrepII-HA-Beclin 1-MITO did not alter the morphology or subcellular localization of ER or mitochondria, respectively.

**Figure 2:**
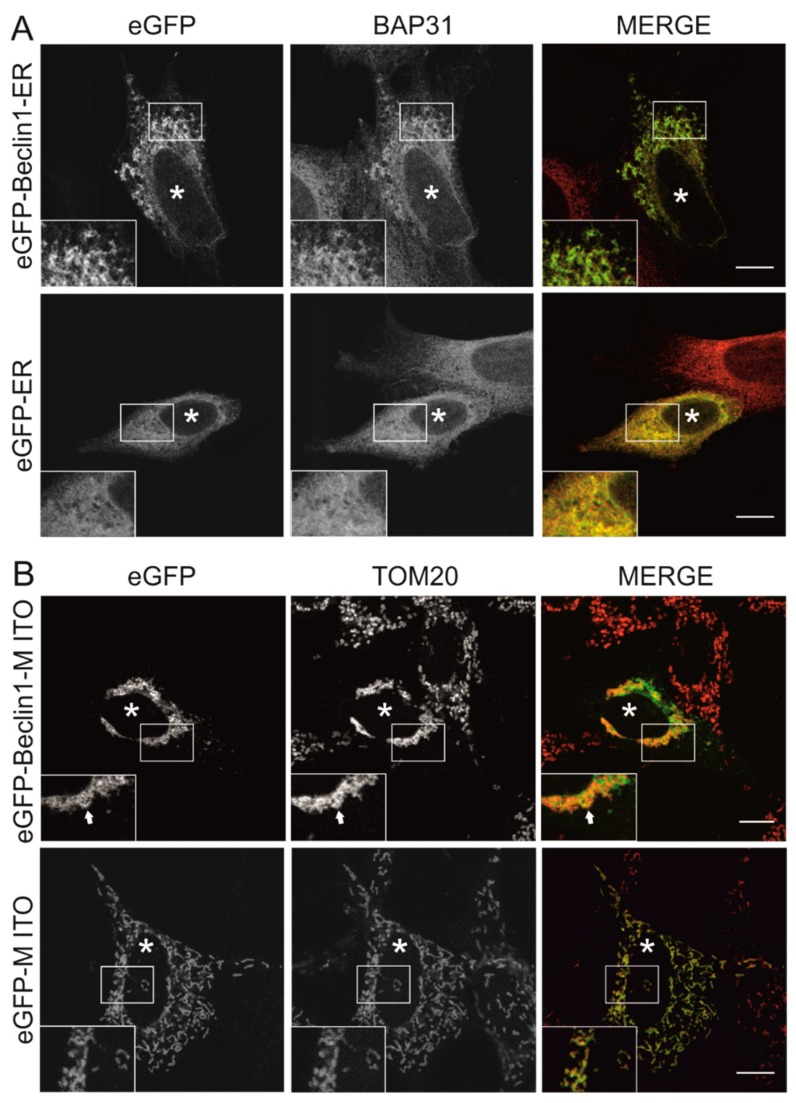
Subcellular localization of eGFP-Beclin 1-ER and eGFP-Beclin 1-MITO, as well as the corresponding targeted control constructs, in mouse embryonic fibroblasts-wild type (MEF-WT). (**A**) MEF-WT cells were transfected with eGFP-Beclin 1-ER or eGFP-ER as indicated, and labelled against endoplasmic reticulum (ER) marker BAP31. (**B**) MEF-WT cells were transfected with eGFP-Beclin 1-MITO and eGFP-MITO as indicated, and labelled against mitochondrial marker TOM20. The arrows indicate one of the structures showing colocalization. Images were taken with a confocal microscope and one optical section is shown. Asterisks indicate cells expressing the constructs. Scale bars, 10 μm.

We also transiently transfected the eGFP-tagged Beclin 1 contructs to MEF cells and used immunostaining to investigate the efficiency of the organelle targeting of these constructs. The targeted Beclin 1 constructs all contained eGFP tag in the N-terminus of Beclin 1, while the peptides for subcellular targeting were in the C terminus of Beclin 1, similar to the constructs used for HEK293 cells (Figure 1A). The constructs are referred to as eGFP-Beclin 1-ER (ER-targeted Beclin 1) and eGFP-Beclin 1-MITO (mitochondrial targeted Beclin 1). We also generated targeting control constructs that did not contain Beclin 1 sequence but only eGFP and the organelle targeting sequence. These constructs are referred to as eGFP-ER (ER-targeted control construct) and eGFP-MITO (mitochondrial targeted control construct). To confirm the subcellular localization of eGFP-Beclin 1-ER we performed immunofluorescence staining with antibodies against BAP31 in wild type MEF cells (MEF-WT). eGFP-Beclin 1-ER (Figure 2A, upper panel) and eGFP-ER (Figure 2A, lower panel) both substantially colocalized with BAP31 as expected. eGFP-Beclin 1-ER and eGFP-ER also showed colocalization with calreticulin, another ER marker protein (Figure S2A), but less colocalization with the soluble ER protein PDI (protein disulphide isomerase, Figure S2B). No colocalization was observed with the Golgi marker GM130 (Figure S2C). Of note, unlike the stable expression of Twin-StrepII-HA-Beclin 1-ER in HEK293 cells, transient expression of eGFP-Beclin 1-ER in MEF changed the morphology of the ER, while eGFP-ER had no effect (Figure 2A). In order to study the ER morphology at high magnification, we performed correlative light electron microscopy (CLEM) with the cells expressing eGFP-Beclin 1-ER.

**Figure 3:**
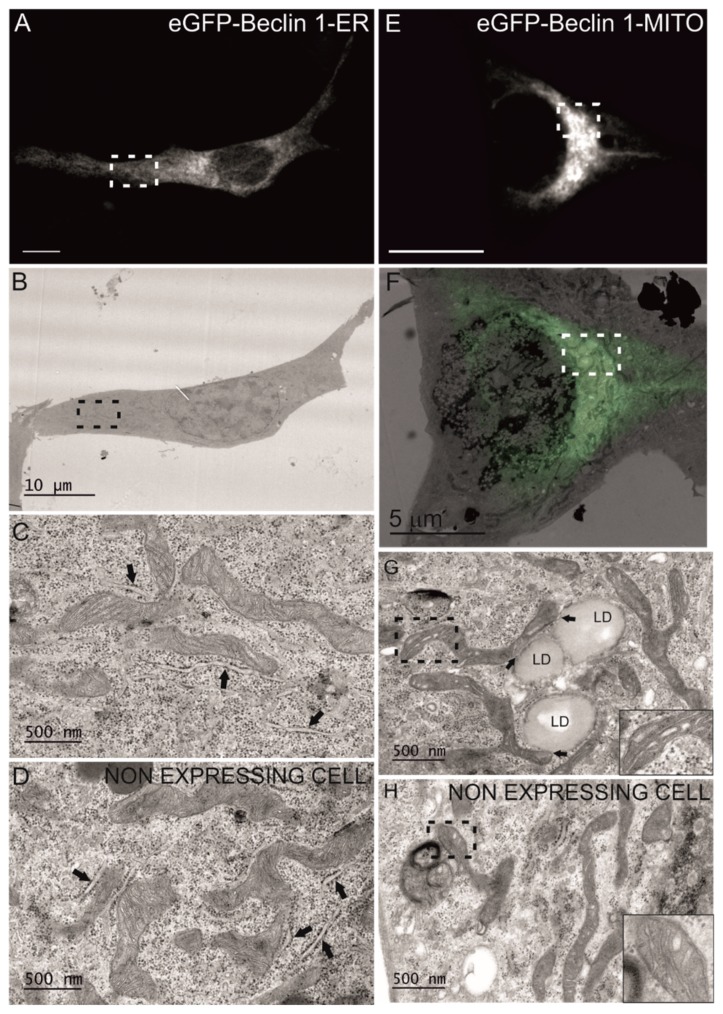
Electron microscopy analysis of MEF-WT cells expressing eGFP-Beclin 1-ER or eGFP-Beclin 1-MITO. The cells were starved for 1 h in amino-acid-free medium before fixation. (**A**–**D**) MEF-WT cells transfected with eGFP-Beclin 1-ER. (**A**) Confocal image of a cell expressing eGFP-Beclin 1-ER. Scale bar, 10 μm. (**B**) Electron micrograph of the same cell as in panel A. (**C**) Higher magnification of the boxed area in panel B, showing normal morphology of rough ER (arrows). (**D**) Electron micrograph of a non-expressing cell from the same sample, showing the morphology of rough ER (arrows). (**E**–**H**) MEF-WT cells transfected with eGFP-Beclin 1-MITO. (**E**) Confocal image of a MEF-WT cell expressing eGFP-Beclin 1-MITO. Scale bar, 10 μm. (**F**) Electron micrograph of the same cell as in panel E. (**G**) Higher magnification of the boxed area in panel F, showing mitochondria and contact sites between lipid droplets (LD) and mitochondria (arrows). (**H**) Electron micrograph of a non-expressing cell from the same sample, showing the morphology of mitochondria. Inserts in panels G and H show mitochondrial cristae at higher magnification.

The cells were seeded on gridded glass bottom, transfected with eGFP-Beclin 1-ER construct, starved for 1 h and processed for CLEM. Fluorescence microscopy was used to locate cells expressing the eGFP-tagged construct, and these same cells were then traced in the electron microscopy sections (Figure 3A–D). The analysis showed that the ultrastructue of the rough ER was normal (Figure 3C), despite the altered pattern observed in part of the eGFP-Beclin 1-ER expressing cells in fluorescence microscopy (Figure 2A). Non-expressing cells from the same sample were imaged for comparison (Figure 3D). Rough ER morphology was similar in cells expressing eGFP-Beclin 1-ER and in non-expressing cells.

To confirm the subcellular localization of eGFP-Beclin 1-MITO in transiently transfected MEF, we performed immunofluorescence with antibodies against TOM20. Both eGFP-Beclin 1-MITO and eGFP-MITO control (Figure 2B) substantially colocalized with TOM20 as expected. Unlike the stable expression of Twin-StrepII-HA-Beclin 1-MITO in HEK293 cells, the transient expression of eGFP-Beclin 1-MITO, but not eGFP-MITO, in MEF had a profound effect on the subcellular localization of mitochondria, causing them to accumulate in the perinuclear area (Figure 2B, upper panel). We quantified this using point counting to estimate the area covered by mitochondrial staining in HeLa cells expressing eGFP-Beclin 1-MITO or eGFP-MITO construct (data not shown). The quantification showed that in cells expressing eGFP-Beclin 1-MITO, 45.8% of the cell area was covered by mitochondria, while in non-expressing cells and eGFP-MITO-expressing cells mitochondria covered 71.6% and 69.8% of the cell area, respectively. We also performed CLEM to study whether mitochondrial morphology was changed in the cells expressing eGFP-Beclin 1-MITO. The analysis showed that mitochondrial ultrastructure was normal despite their clustering around the nucleus (Figure 3E–H). Of note, in cells expressing eGFP-Beclin 1-MITO, mitochondria showed contacts with lipid droplets, but we did not observe signs of lipophagy, nor increased mitophagy (Figure 3G). Non-expressing cells from the same sample were imaged for comparison (Figure 3H). Mitochondrial ultrastructure was similar in cells expressing eGFP-Beclin 1-MITO (Figure 3G) and in non-expressing cells (Figure 3H).

Taken together, these results showed that the Beclin 1-ER and Beclin 1-MITO constructs localized to their expected organelles in stably expressing HEK293 cells and in transiently transfected MEF cells. Moreover, also the eGFP-ER and eGFP-MITO control constructs (with no Beclin 1 insert) both localized to the expected subcellular compartments.

### 3.2. Expression of the Targeted Beclin 1 Constructs Does Not Induce Organelle Stress

Next we assessed whether targeting of Beclin 1 to ER or mitochondria induced ER or mitochondrial stress. ER stress was monitored using immunofluorescence staining of the transcription factor CCAT-enhancer-binding protein homologous protein (CHOP) [42], which is targeted to the nucleus during ER stress. Tunicamycin was used as a positive control, since it induces ER stress by inhibiting N-glycosylation, which causes accumulation of incorrectly folded proteins in the ER. MEF-WT cells expressing the eGFP-Beclin 1-ER or eGFP-ER were starved of serum and amino acids without or with tunicamycin, fixed and stained with anti-CHOP. No CHOP was detected in the nucleus in cells expressing either of the eGFP-tagged contructs (Figure S3), unless tunicamycin was present. This also applied to the non-expressing cells. These results indicated that targeting eGFP-Beclin 1 to the ER did not induce ER stress. This is in agreement with the CLEM results showing normal ultrastructure of ER in MEF expressing eGFP-Beclin 1-ER (Figure 3C).

Mitochondrial stress was assessed using two methods, by measuring mitochondrial membrane potential and by monitoring OPA1 cleavage. To assess whether transient expression of eGFP-Beclin 1-MITO induced mitochondrial stress in MEF-WT cells, we quantified mitochondrial membrane potential in live cells by using TMRE [43] (Figure S4). Quantification of TMRE signals showed that mitochondrial membrane potential was similar in non-expressing cells and in cells expressing either eGFP-Beclin 1-WT, eGFP-Beclin 1-MITO, or eGFP-MITO (Figure S4B). The uncoupling drug CCCP, used as a positive control, dramatically decreased TMRE staining as expected. The inner mitochondrial membrane GTPase OPA1 is proteolytically cleaved during mitochondrial stress [44]. We assessed OPA1 cleavage in HEK293 cells inducibly expressing Twin-StrepII-HA-Beclin 1-WT or Twin-StrepII-HA-Beclin 1-MITO. No OPA1 cleavage was observed after induction of the expression with tetracycline in either cell line (Figure S5). As expected, CCCP treatment led to fully cleaved OPA1 in all cell lines tested. Taken together, these results confirmed that targeting Beclin 1 to mitochondria did not induce mitochondrial stress. These findings are in agreement with the CLEM results showing normal mitochondrial ultrastructure in MEF transiently expressing eGFP-Beclin 1-MITO (Figure 3G).

### 3.3. The Targeted Beclin 1 Constructs Bind to Known Autophagy-Related Interactors of Beclin 1

**Figure 4:**
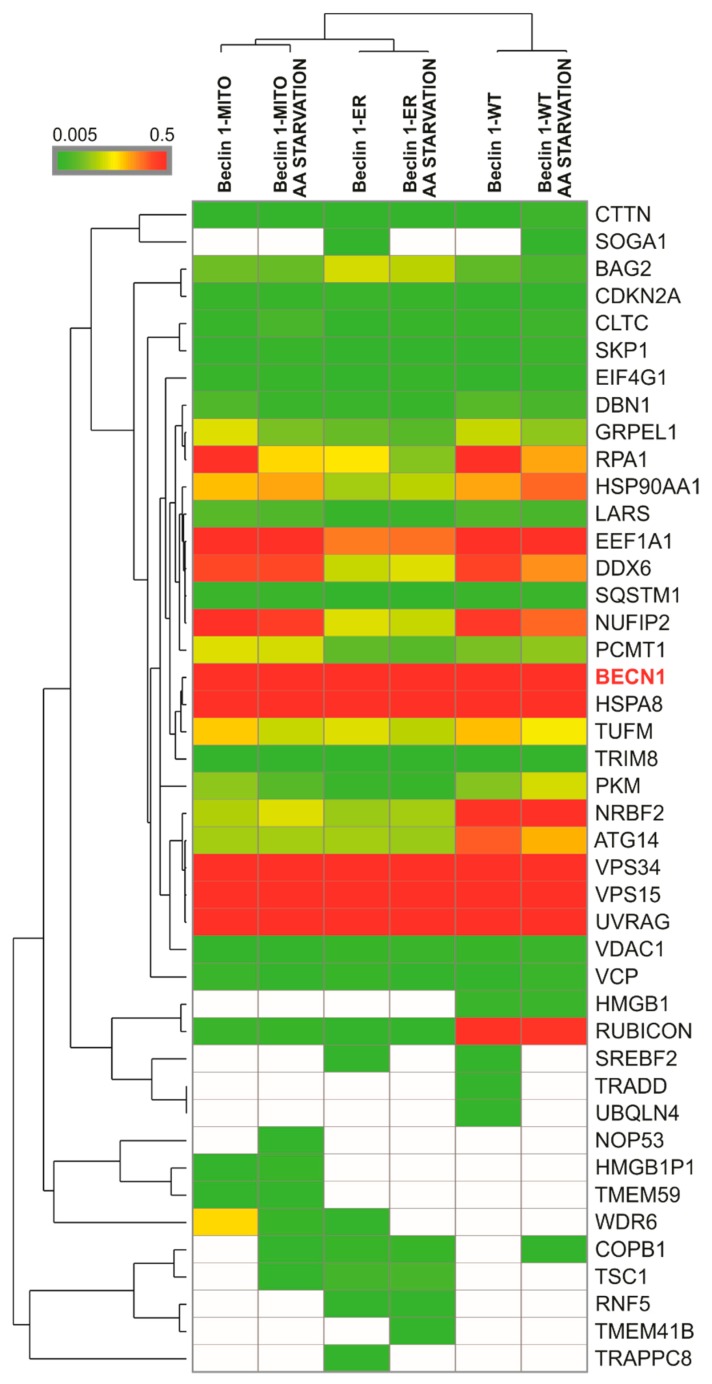
Heat map with hierarchical clustering of the interactors identified for the Beclin 1 constructs by affinity purification–mass-spectrometry (AP–MS). Stable HEK293 cell lines inducibly expressing Twin-StrepII-HA-tagged Beclin 1 constructs were induced by adding 2 μg/mL tetracycline to the culture medium for 24 h, kept in full medium or starved in amino-acid (AA) free medium for 2 h. The interacting proteins of Beclin 1 were purified using Strep-Tactin beads and the purified proteins were analyzed using mass spectrometry. Heat map shows a summary of the interacting proteins of the Beclin 1 constructs. The colours correspond to protein-relative abundance, using normalized spectral counts (Beclin 1 bait abundance is set to 1). On the bait-normalized spectral count scale, red and shades of red correspond to abundant interactions between Beclin 1 constructs and the hits, while green and shades of green correspond to less abundant interactions. White colour indicates barely detectable or non-detectable interaction. Values are averages of three biological replicates.

To verify that the Beclin 1-ER and Beclin 1-MITO constructs are still able to bind the known autophagy-associated binding partners of Beclin 1, we performed affinity-purification followed by mass spectrometry using the stable HEK293 cell lines expressing the targeted Beclin 1 constructs. We used both non-starved and amino-acid starved cells in these experiments [5,13,45,46]. Cells expressing Twin-StrepII-HA-Beclin 1-WT, Twin-StrepII-HA-Beclin 1-ER or Twin-StrepII-HA-Beclin 1-MITO were induced with tetracycline for 24 h prior to the experiments. Beclin 1 complexes were purified from cell extracts using Strep-Tactin beads and analyzed by mass spectrometry. The results are summarized as a heat map in Figure 4, and the data is presented as Supplementary Table S2. Our results revealed a number of high-confidence interacting proteins (HCIPs) for the Beclin 1 constructs. The prey proteins are shown with their corresponding bait normalized abundance, and they cluster hierarchically into groups, indicated by the brackets on the left side of the heat map (Figure 4). The three Beclin 1 constructs were also clustered in order to show their interaction profiles. The results showed that Beclin 1-ER and Beclin 1-MITO formed expected molecular interactions similar to Beclin 1-WT.

The Beclin 1 complex involved in autophagosome formation, known as complex I, contains Beclin 1, ATG14, the class III PI3-kinase (PI3KC3) VPS34, and the regulatory serine-threonine kinase VPS15 that plays a central role in the assembly of the Beclin 1 complex and in the regulation of VPS34 activity [12]. ATG14 is also known as Beclin 1-Associated Autophagy-Related Key Regulator (Barkor) [13]. Beclin 1 can also form a complex with UVRAG (UV radiation resistance-associated gene) which participates in endosome-autophagosome fusion [47]. Another component of the Beclin 1-VPS34-UVRAG complex is Rubicon, also known as the Beclin 1-associated RUN domain-containing protein (Baron). Rubicon has been shown to be a negative regulator of autophagy [45].

Our results (Figure 4, Supplementary Table S2) showed that Beclin 1-WT, Beclin 1-ER and Beclin 1-MITO all made abundant interactions with VPS34 and VPS15. These two interactions were the most abundant in our analysis, in agreement with the consensus that Beclin 1, VPS34 and VPS15 form the core Beclin 1 complex needed for autophagosome biogenesis [48]. All Beclin 1 constructs interacted at equal high abundance with UVRAG. Beclin 1-WT made abundant stable interactions with ATG14, while Beclin 1-MITO and Beclin 1-ER formed less abundant but still detectable interactions. Further, all Beclin 1 constructs showed interaction with nuclear receptor binding factor 2 (NRBF2) [46]. Beclin 1-WT formed more abundant interactions with Rubicon, compared with Beclin 1-ER and Beclin 1-MITO. With the exception of a modest decrease in the interaction of Beclin 1-WT with ATG14, amino-acid starvation did not dramatically affect any of the autophagy-relevant interactions in our analysis.

To summarize, our data showed that all Beclin 1 constructs were found in a complex with known interactors of Beclin 1 that are required for autophagosome formation. However, compared to Beclin 1-ER and Beclin 1-MITO, Beclin 1-WT showed more interaction with Rubicon, which is involved in autophagy inhibition.

### 3.4. Effect of Beclin 1 Targeting on Autophagy in Human Embryonic Kidney (HEK293) Cells

Western blotting was used to compare the expression levels of the Twin-StrepII-HA tagged Beclin 1 constructs in the stable HEK293 cell lines. The stable inducible expression levels of the Beclin 1 constructs showed some variation, likely due to the toxicity of the overexpression, and possibly also reflecting the different size of the available targeted cell compartments: the surface area of ER is much larger than the surface area of outer mitochondrial membranes [49]. Beclin 1-ER showed the highest expression level, while Beclin 1-WT and Beclin 1-MITO constructs showed similar lower expression levels (Figure S6A,B). We also used Western blotting to check whether the targeting of Beclin 1 to ER or mitochondria had an effect on the levels of ER or mitochondrial proteins (data not shown). Compared to parental HEK293 cells, all Beclin 1 constructs caused a similar increase in the levels of the ER protein BAP31 (211%, 226% and 193% for WT, ER and MITO Beclin 1, respectively). On the contrary, all Beclin 1 constructs caused a decrease in the levels of the mitochondrial protein TOM20 (34%, 31% and 56% for WT, ER and MITO Beclin 1, respectively). These results suggest that the altered expression levels of BAP31 and TOM20 were caused by increased Beclin 1 levels rather than Beclin 1 targeting.

**Figure 5:**
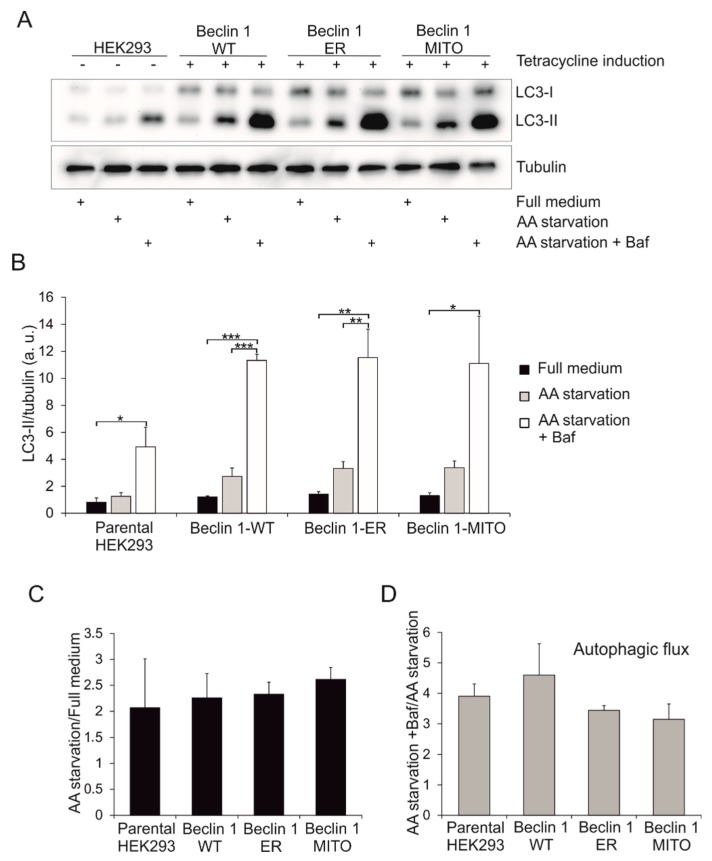
Effect of Beclin 1 constructs on LC3 levels in stable inducible HEK293 cells. HEK293 cells stably expressing Twin-StrepII-HA-tagged Beclin 1 constructs were induced by adding 2 μg/mL tetracycline to the culture medium for 24 h. Normal HEK293 cells were used as control. Cells were kept in full medium or starved in amino-acid (AA) free medium for 2 h with or without 100 nM bafilomycin A1. (**A**) Cell lysates were analyzed by Western blotting with LC3 antibody. Tubulin was used as loading control. (**B**) Relative LC3-II levels (LC3-II/tubulin). (**C**) LC3-II relative increase during starvation (AA starvation/Full medium). (**D**) Autophagic flux (AA starvation+Baf/AA starvation). Results are shown as mean of three biological replicas (N = 3). Error bars represent SEM. One-way analysis of variance (ANOVA) followed by Tukey Kramer post hoc test was used to assess statistical significance. * *p* < 0.05, ** *p* < 0.01, *** *p* < 0.001.

In order to study the effects of the targeted Beclin 1 constructs on autophagy in the stable HEK293 cell lines, we monitored LC3 lipidation using Western blotting. The same samples that were run to test the expression levels of the HA-tagged Beclin 1 constructs were used in these experiments. Three different treatments were used: full medium, serum and amino acid starvation for 2 h (AA starvation), and serum and amino acid starvation with bafilomycin A1 for 2 h (AA starvation + Baf). Bafilomycin A1 blocks the lysosomal proton pump, and thus it prevents the maturation and clearance of autophagosomes, revealing information on autophagic flux. Compared to parental HEK293 cells, stable expression of all Beclin 1 constructs increased the levels of the autophagosome-associated form of LC3, LC3-II, in both full medium and AA starvation (Figure 5A,B).

In order to quantify the extent to which AA starvation increased the LC3-II levels, we divided the relative LC3-II levels (LC3-II/tubulin) under AA starvation with the levels in full medium (Figure 5C). This calculation revealed no statistically significant differences between the four samples. To get an estimation of autophagic flux during AA starvation, we divided the relative LC3-II levels in AA starvation in the presence of bafilomycin A1 with the LC3-II levels in AA starvation only (Figure 5D). This normalization approach revealed that compared to parental HEK293 cells, only Beclin 1-WT slightly increased the autophagic flux. However, the differences did not reach statistical significance (Figure 5D). These results therefore suggested that stable overexpression of none the Beclin 1 constructs could significantly alter autophagic flux.

We also performed Western blotting of the autophagy adaptor and cargo protein SQSTM1/p62 on these same samples. In agreement with earlier published results for normal HEK293 cells [50], the levels of p62 in parental HEK293 did not dramatically change during the three experimental treatments (Figure S7A). Compared to the parental HEK293 cells, none of the Beclin 1 constructs significantly altered the p62 levels (Figure S7B). These results were in agreement with the LC3-II Western blot data, indicating relatively normal autophagic flux in HEK293 cells despite overexpression of the Beclin 1 constructs.

### 3.5. Effects of Beclin 1 Targeting on Autophagy in Wild-Type and ULK1/ULK2 Double Knockout Mouse Embryonic Fibroblasts (MEFs)

Next we investigated how forced targeting of Beclin 1 affects autophagosome formation in ULK1 and ULK2 double knockout mouse embryonic fibroblasts (MEF-ULK1/2-KO). The rationale behind these experiments was to investigate whether targeting of Beclin 1 to autophagosome assembly site was the main role of the ULK1/2 kinases during autophagosome biogenesis. It has been shown that in the absence of ULK1 and ULK2, autophagy induction by amino acid starvation is impaired [17]. After starvation, the number of LC3 puncta in MEF-ULK1/2-KO cells was reduced to approximately half of the amount in wild-type cells. Our experiments were done using one of the MEF-ULK1/2-KO cell lines described in McAlpine et al. [17].

**Figure 6:**
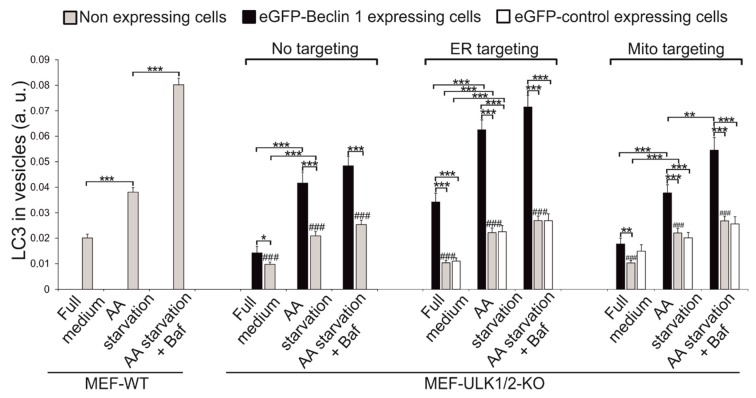
Quantification of LC3 labelling in MEF-WT and MEF ULK1/2-KO cells expressing the eGFP-tagged constructs. MEF-ULK1/2-KO cells were transfected with eGFP-Beclin 1-WT, eGFP-Beclin 1-ER, eGFP-ER, eGFP-Beclin 1-MITO, or eGFP-MITO. Cells were kept in full medium or starved in amino-acid (AA) free medium for 1 h with or without 100 nM bafilomycin A1, fixed and labelled with LC3 antibody. CellProfiler was used to quantify the proportion of LC3 in vesicles, both in cells expressing the eGFP-tagged constructs and in non-expressing cells in the same sample. Results are shown as mean and SEM of 50 eGFP-tagged construct expressing cells and 150 non expressing cells (N = 50 and N = 150, respectively). One-way ANOVA followed by Tukey Kramer post hoc test was used to test statistical significance. * *p* < 0.05, ** *p* < 0.01, *** *p* < 0.001 for comparisons within cell lines. ^###^ *p* < 0.001 for non-expressing MEF-ULK1/2-KO cells compared to non-expressing MEF-WT cells under the same condition.

We studied whether forced targeting of Beclin 1 could rescue autophagosome formation in cells lacking the ULK1/ULK2 signalling complex. We used a microscopic autophagy assay on transiently transfected cells, in order to avoid the effects of selective cloning on cell functions. This approach also made it possible to use the non-expressing neighbouring cells in the same samples as an internal control in the microscopic autophagy assay. We used immunofluorescence of endogenous LC3 to monitor autophagosome formation. Instead of monitoring the number of LC3 puncta per cell, we used the relative amount of LC3 signals originating from vesicles (vesicular LC3) as a measure of autophagosome accumulation. In order to obtain these values, the intensity of LC3 signals originating from vesicles was divided by the total amount of LC3 signals in the same cell. The quantification was done for both cells expressing the eGFP-tagged constructs, and for non-expressing cells on the same coverslip as an internal control. Autophagosome clearance was blocked by addition of bafilomycin A1. In MEF-WT cells, amino acid starvation increased vesicular LC3, and addition of bafilomycin A1 to the starvation medium increased vesicular LC3 further, as expected (Figure 6, Figure S8).

MEF ULK1/2-KO cells were transfected with the eGFP-Beclin 1 and control constructs and starved (with or without bafilomycin A1) for 1 h (Figure 6, Figures S9–S12). Overexpression of the non-targeted eGFP-Beclin 1-WT was able to significantly rescue generation of LC3-positive autophagosomes in MEF-ULK1/2-KO cells as compared to non-expressing cells (Figure 6, compare black and grey columns, Figure S9). Under amino acid starvation, the amount of vesicular LC3 was similar in ULK1/2-KO cells expressing Beclin 1-WT and in MEF-WT cells (Figure 6). However, unlike in MEF-WT, addition of bafilomycin A1 only marginally increased the amount of vesicular LC3 in ULK1/2-KO cells, suggesting the autophagosomes formed in the ULK1/2-KO cells did not flux efficiently.

Next we studied the ability of ER-targeted Beclin 1 to rescue autophagosome formation in ULK1/2-KO MEFs. Expression of eGFP-Beclin 1-ER significantly increased the formation of autophagosomes both under basal and AA starvation conditions (Figure 6, Figures S10 and S11). Under amino-acid starvation, the level of vesicular LC3 was even higher than in MEF-WT, and reached nearly ~90% of MEF-WT when bafilomycin A1 was present. However, similar to the eGFP-Beclin 1-WT, addition of bafilomycin A1 did not significantly increase vesicular LC3, suggesting the autophagosomes formed in the ULK1/2-KO cells did not flux efficiently. We also confirmed that expression of eGFP-ER had no effect on amounts of vesicular LC3 (Figure 6, compare white and grey bars, Figure S11).

Finally, we investigated the ability of eGFP-Beclin 1-MITO to restore autophagosome formation in the ULK1/2-KO MEFs (Figure 6, Figures S10 and S12). Expression of eGFP-Beclin 1-MITO had a similar effect on vesicular LC3 as eGFP-Beclin 1-WT. Under amino acid starvation, cells expressing eGFP-Beclin 1-MITO contained similar amount of vesicular LC3 than MEF-WT. Addition of bafilomycin A1 significantly increased vesicular LC3, although not as efficiently as in MEF-WT. This suggested that autophagosomes formed in the ULK1/2-KO cells expressing eGFP-Beclin 1-MITO were able to flux to some degree. Finally, we confirmed that eGFP-MITO did not have any effect on vesicular LC3 in any of the experimental conditions (Figure 6, Figure S12).

**Figure 7:**
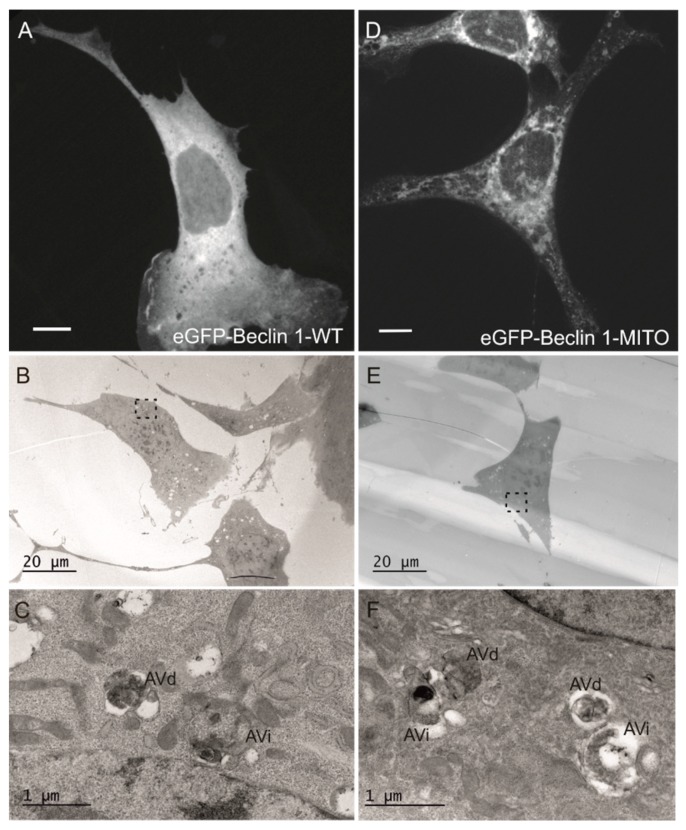
Electron microscopy analysis of autophagosomes in MEF-ULK1/2-KO cells expressing eGFP-Beclin 1-WT or eGFP-Beclin 1-MITO. The cells were transfected with eGFP-Beclin 1 constructs and starved in amino-acid free medium for 1 h before fixation. (**A**–**C**) eGFP-Beclin 1-WT. (**A**) Confocal image of a cell expressing eGFP-Beclin 1-WT. Scale bar, 10 μm. (**B**) Electron micrograph of the same cell as in panel A. (**C**) Higher magnification of the boxed area in B, showing AVi (initial autophagic vacuoles/autophagosomes) and AVd (degradative autophagic vacuoles). (**D**–**F**) eGFP-Beclin 1-MITO. (**D**) Confocal image of a cell expressing eGFP-Beclin 1-MITO. Scale bar, 10 μm. (**E**) Electron micrograph of the same cell as in panel D. (**F**) Higher magnification of the boxed area in E, showing AVi and AVd.

**Figure 8:**
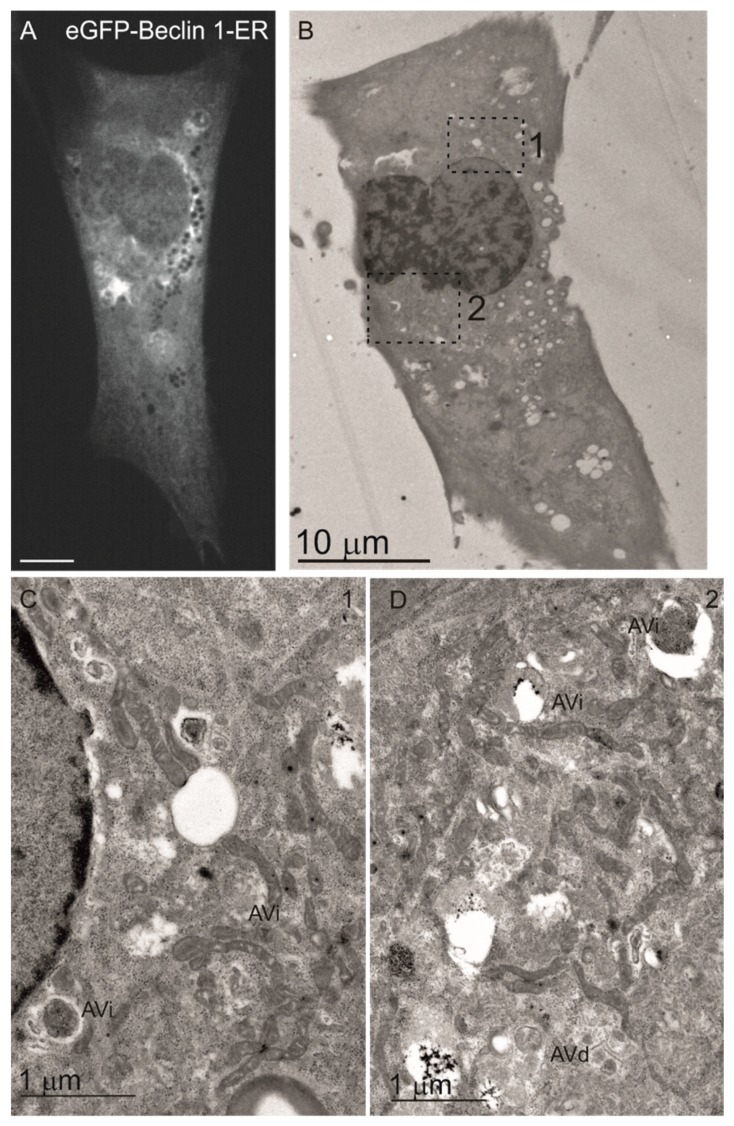
Electron microscopy analysis of ULK1/2-KO MEFs expressing eGFP-Beclin 1-ER. The cells were transfected with eGFP-Beclin 1-ER and starved in amino-acid free medium for 1 h before fixation. (**A**) Confocal image of a cell expressing eGFP-Beclin 1-ER. Scale bar, 10 μm. (**B**) Electron micrograph of the same cell. (**C**,**D**) Higher magnification images of boxed areas in B, showing normal morphology of AVi (initial autophagic vacuoles/autophagosomes) and AVd (degradative autophagic vacuoles).

To further confirm these results, and to study the ultrastructure of the autophagosomes in MEF-ULK1/2-KO cells expressing the Beclin 1 constructs, we used CLEM. Electron microscopy showed that MEF-ULK1/2-KO cells expressing eGFP-Beclin 1-WT or eGFP-Beclin 1-MITO (Figure 7) contained autophagic structures whose morphology was identical to autophagic structures in normal amino-acid starved cells [51]. Similar observations were done for MEF-ULK1/2-KO cells expressing eGFP-Beclin 1-ER (Figure 8).

To conclude, these results suggested that expression of Beclin 1 can rescue autophagosome formation in cells lacking the ULK1/ULK2 autophagy initiation complex. Moreover, Beclin 1 targeted to the ER was the most effective in stimulating autophagosome formation. Consistent with this robust rescue effect, expression of Beclin 1-ER was able to stimulate autophagosome formation significantly even under full nutrient conditions which normally lead only to basal levels of autophagy. Upon addition of bafilomycin A1 to the starvation medium, MEF-ULK1/2-KO cells expressing the Beclin 1 constructs accumulated significantly less vesicular LC3 than MEF-WT cells, indicating that the Beclin 1 constructs were not able to rescue the autophagy flux in MEF-ULK1/2-KO cells to the level observed in MEF-WT cells. Taken together, our results showed that overexpression of Beclin 1 can partially rescue autophagosome formation in cells lacking any function of the ULK1/ULK2 complex. More specifically, while overexpression of Beclin 1-WT or Beclin 1-MITO was sufficient to provide partial rescue, targeting of Beclin 1 to the ER provided a more robust signal to activate autophagosome formation.

### 3.6. Wild-Type Beclin 1 Is Enriched in the Endoplasmic Reticulum (ER) during Amino Acid Starvation

**Figure 9:**
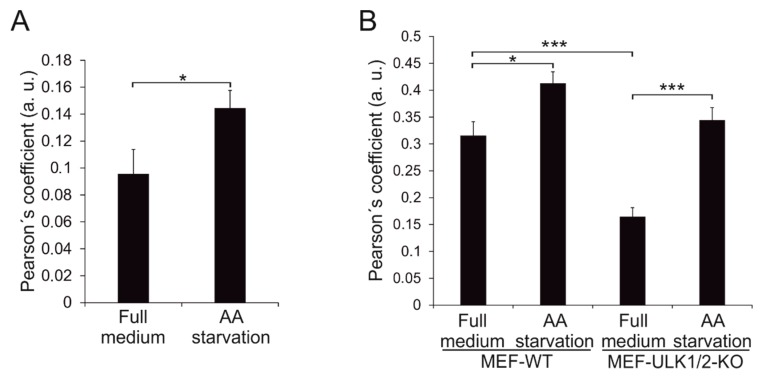
Quantitaive analysis of colocalization for Beclin 1-WT with ER markers in non-starved and amino-acid starved HEK293 and MEF cells. (**A**) Colocalization of Beclin 1-WT and calreticulin in HEK293 cells inducibly expressing Twin-StrepII-HA-Beclin 1-WT. The cells were induced with 2 μg/mL tetracycline for 24 h, either kept in full medium or starved in amino-acid (AA) free medium for 1 h, fixed and labelled with anti-HA and anti-calreticulin. Imaris 9.2 was used to quantify the colocalization between Beclin 1-WT and calreticulin on confocal microscopy images (Figure S13). Results are shown as mean of Pearson´s correlation coefficient and standard error of the mean (SEM), calculated using 25 images, each containing 2 to 6 cells. A two-tailed unpaired t-test was used to test statistical significance. * *p* < 0.05. (**B**) Colocalization of Beclin 1-WT and BAP31 in MEF-WT and MEF-ULK1/2-KO cells transiently expressing eGFP-Beclin 1-WT. The cells were either kept in full medium or starved in amino-acid (AA) free medium for 1 h, fixed and labelled with anti-BAP31. Imaris 9.2 was used to quantify the colocalization between Beclin 1-WT and BAP31 on confocal microscopy images (Figure S14). The results are shown as mean of Pearson´s correlation coefficient and SEM for 20 cells. One-way ANOVA and Tukey Kramer post hoc test were used to test statistical significance. * *p* < 0.05, *** *p* < 0.001.

Next, we studied whether wild type Beclin 1 becomes enriched on the ER during amino acid starvation that causes a robust induction of autophagy. HEK293 cells inducibly expressing Twin-StrepII-HA-Beclin 1-WT were induced with tetracycline for 24 h, and then either kept in full medium or starved for serum and amino acids for 1 h. The cells were fixed and stained with anti-HA and anti-calreticulin, which gave clearest ER staining in HEK293 cells. Colocalization of Beclin 1 and the ER marker was estimated using Imaris software. Wild-type Beclin 1 showed a limited colocalization with ER in full medium (Figure 9A, Figure S13). However, we observed a 1.5-fold increase in the Pearson’s coefficient after amino acid starvation. This suggested that Beclin 1 was enriched on the ER during autophagy induction by amino acid starvation.

Finally, we used MEF-WT and MEF-ULK1/2-KO cells to investigate the role of ULK1/2 in the ER enrichment of Beclin 1. Cells transfected with eGFP-Beclin 1-WT were either kept in full medium or starved for serum and amino acids for 1 h, fixed and stained with anti-BAP31, which gave the clearest ER staining in MEF cells. Colocalization of Beclin 1 and the ER marker was estimated as above. Beclin 1-WT showed limited colocalization with ER in full medium, and remarkably, the Pearson’s correlation coefficient was reduced to half in ULK1/2-KO cells (Figure 9B, Figure S14). In both MEF-WT and MEF-ULK1/2-KO, the colocalization significantly increased during amino-acid starvation. These results suggested that a pool of Beclin 1 was maintained on the ER during basal conditions, and that this was regulated by ULK1/2. Moreover, localization of Beclin 1 to the ER was stimulated by amino-acid starvation, and this was regulated by additional ULK1/2-independent pathways.

## 4. Discussion

Beclin 1 holds a pivotal role in autophagosome biogenesis since it binds to the class III PI3-kinase VPS34 whose activity is indispensable for the process [9,10]. Despite its importance, the exact role of Beclin 1 subcellular localization in autophagosome formation is still unclear. In this study, we generated differentially targeted Beclin 1 constructs to study how changes in the subcellular localization of Beclin 1 affect autophagosome biogenesis. We also studied whether forced targeting of Beclin 1 to the endoplasmic reticulum or mitochondria could rescue autophagosome formation in the absence of upstream signalling from the ULK1 and ULK2 kinases.

Beclin 1 is found in a complex with several partners that are essential for autophagosome formation. In our study, the assembly of the Beclin 1 complex was confirmed by affinity-purification mass-spectrometry. VPS34 and VPS15 made abundant interactions with all our Beclin 1 constructs, in agreement with previous literature indicating them as indispensable for autophagosome formation [10,11,12]. UVRAG also made abundant interactions with all the Beclin 1 constructs. Earlier studies have set UVRAG as an essential autophagy checkpoint due to its dual role in the process. UVRAG binds to Beclin 1 and mediates autophagosome maturation [47]. A previous study showed that UVRAG can also enhance the interaction between Beclin 1 and VPS34, thus increasing the kinase activity and promoting autophagosome formation [52]. Our study also showed that Rubicon made strongest interactions with wild-type Beclin 1, but less abundant interactions with mitochondrial-targeted and ER-targeted Beclin 1. Since Rubicon is an autophagy inhibitory protein [45], our findings may suggest that the inhibitory interaction between Rubicon and Beclin 1 preferentially occurs in other locations than mitochondria and the ER. Of note, Ambra 1, another Beclin 1 interactor for autophagy induction [53], was not observed in our study among Beclin 1 interacting proteins. A possible explanation for this could be the transient nature of Ambra 1 interactions with Beclin 1, in contrast to more stable interactions with other binding partners.

We studied how targeting of Beclin 1 to the ER or mitochondria affected autophagosome biogenesis in ULK1/ULK2 double knockout MEF cells that have been shown to be defective in this process [17]. ER-targeted Beclin 1 showed the strongest ability to induce autophagosome formation in ULK1/ULK2 double knockout cells, while the wild type and the mitochondrial targeted Beclin 1 showed a similar smaller rescue effect. Several previous studies have also shown that autophagy can be induced independently of ULK1/ULK2. One study showed that ATG13, a subunit of the ULK1/ULK2 complex, was necessary for autophagy induction in DT40 chicken cells [54]. This study showed that ATG13 function relies on its interaction with another component of the ULK complex, FIP200, and that knockout of ATG13 blocked autophagy induction. Interestingly, double knockout of ULK1 and ULK2 did not have the ATG13 knockout phenotype. Thus these findings suggest a non-essential role for ULK1/ULK2 in autophagy induction. Another study showed that Leucine-Rich Repeat Kinase 2 (LRRK2) is involved in non-canonical regulation of autophagy in astrocytic cells [55]. This study demonstrated that chemical inhibition of LRRK2 induced autophagosome formation. More importantly, this was independent of ULK1 activity but dependent on the Beclin1/VPS34 complex. Two other separate studies have also shown that ULK1/ULK2 signalling is dispensable for the initiation of events that lead to autophagosome formation [56,57]. These results all support our findings that Beclin 1 is sufficient to promote autophagosome formation in the absence of upstream signalling from ULK1/ULK2.

Beclin 1 itself can regulate autophagosome formation through protein-protein interactions or post-translational modifications. In human erythroleukemia K562 cells and in rat hepatoma H4IIE cells, Beclin 1 is regulated by a non-canonical MEK/ERK module (mitogen-activated protein kinase/extracellular signal-regulated kinase kinase; extracellular signal-regulated kinase) downstream of the AMP-activated protein kinase (AMPK) [58]. This study showed that, upon autophagy stimulation, the activated MEK/ERK module upregulates Beclin 1, thus modulating autophagy levels. Another study showed that the interaction between Beclin 1 and Vacuole Membrane Protein 1 (VMP1) regulates autophagosome formation in HeLa cells [59]. Finally, a range of data indicates that Beclin 1 post-translational modifications such as phosphorylation and ubiquitination can be important during the regulation of autophagy in response to various stimuli [60].

In our study, Beclin 1 targeted to the ER was most effective in inducing autophagosome formation in ULK1/2 knockout MEFs, while Beclin 1 targeted to mitochondria and wild-type Beclin 1 showed a similar, smaller rescue effect. This is in agreement with several previous studies that set the ER as the site for autophagosome biogenesis [19,20,23,61]. A central dogma in the autophagy field is that autophagosomes emerge in omegasomes, an ER subdomain enriched with PI3P [21].

Our results showed that while Beclin 1 overexpression in MEF-ULK1/2-KO cells was able to rescue autophagosome formation, the maturation of autophagic structures was less efficiently recovered, as shown by the minimal additional increase of vesicular LC3 upon addition of bafilomycin A1 to the starvation medium. Interestingly, previous studies show that in addition to the suggested roles in autophagosome biogenesis, ULK1/ULK2 are involved in later stages of the autophagy pathway. Beclin 1-VPS34-VPS15 core complex forms two different complexes that are involved in separate stages of autophagy [5,47]. One Beclin 1-VPS34 complex contains ATG14 that regulates autophagy induction [5], and the second Beclin 1-VPS34 complex contains UVRAG, but not ATG14, and controls endocytic trafficking and autophagosome maturation [47]. Upon starvation, ULK1 phosphorylates Beclin 1 when it is bound to ATG14, thus enhancing the activity of the VPS34 kinase and promoting autophagosome formation. Furthermore, ULK1 also phosphorylates Beclin 1 when it is bound to UVRAG, thus regulating autophagosome maturation [14]. In the absence of the ULK1/ULK2, Beclin 1 phosphorylation is abnormal, which likely impairs autophagosome maturation as observed in our study. Another recent study investigated the role of the ULK1 complex at the later stages of autophagy pathway [62]. ULK1 directly binds syntaxin 17 (STX17) and modulates its interaction with synaptosomal-associated protein 29 (SNAP29), thus allowing autophagosome-lysosome fusion. These findings could also explain the inefficient autophagy flux we observed with the Beclin 1 constructs in ULK double knockout cells.

Finally, we showed that wild-type Beclin 1 is enriched in the ER during amino-acid starvation that robustly induces autophagy. Under basal conditions, but not during amino-acid starvation, the ER enrichment was reduced in the ULK1/2-KO cells, suggesting that ULK kinases may play a role in the ER enrichment of Beclin 1 under basal conditions.

To summarize, our results are in agreement with the crucial role of Beclin 1 in autophagosome biogenesis. We demonstrated that Beclin 1 was able to sustain autophagosome formation in the absence of the ULK1/ULK2 kinases, most efficiently when targeted to the ER. Our results thus support the idea that autophagosome formation can proceed independently of ULK1 and ULK2. Moreover, our data support the view that the ULK1/ULK2 kinases might play an important role in autophagosome maturation to degradative autolysosomes, as well as in the enrichment of Beclin 1 in ER under basal conditions.

## Supplementary Materials

The following are available online at https://www.mdpi.com/2073-4409/8/5/475/s1: Figure S1: Subcellular localization of wild-type Beclin 1 in HEK293 cells inducibly expressing Twin-StrepII-HA-tagged Beclin 1; Figure S2: Colocalization of eGFP-Beclin 1-ER and eGFP-ER with ER markers calreticulin and protein disulphide isomerase (PDI), and Golgi marker GM130, in MEF-WT cells; Figure S3: Expression of eGFP-Beclin 1-ER in MEF-WT cells does not induce ER stress; Figure S4: Expression of eGFP-Beclin 1-MITO in MEF-WT cells does not induce mitochondrial stress as detected using tetramethylrhodamine ethyl ester (TMRE) to monitor membrane potential; Figure S5: Expression of Twin-StrepII-HA-Beclin 1-MITO in HEK293 cells does not induce mitochondrial stress as detected by OPA1 cleavage; Figure S6: Expression levels of Twin-StrepII-HA-Beclin 1 constructs in HEK293 cells; Figure S7: Effect of Beclin 1 constructs on p62 levels in HEK293 cells stably expressing Twin-StrepII-HA-tagged Beclin 1 constructs; Figure S8: Representative immunofluorescence images for the quantification of vesicular LC3 in non-expressing MEF-WT; Figure S9: Representative immunofluorescence images for the quantification of vesicular LC3 in MEF-ULK1/2-KO cells expressing eGFP-Beclin 1-WT; Figure S10: Higher magnification immunofluorescence images showing vesicular LC3 in MEF-ULK1/2-KO cells expressing eGFP-Beclin 1-ER and eGFP-Beclin 1-MITO; Figure S11: Representative immunofluorescence images for the quantification of vesicular LC3 in MEF-ULK1/2-KO cells expressing eGFP-Beclin 1-ER and eGFP-ER; Figure S12: Representative immunofluorescence images for the quantification of vesicular LC3 in MEF-ULK1/2-KO cells expressing eGFP-Beclin 1-MITO and eGFP-MITO; Figure S13: Representative immunofluorescence images for the quantification of colocalization between Twin-StrepII-HA-Beclin 1-WT and calreticulin in HEK293 cells; Figure S14: Representative immunofluorescence images for the quantification of colocalization between eGFP-Beclin 1-WT and BAP31 in MEF-WT cells; Table S1: List of oligonucleotide primers used for the assembly of the Beclin 1 constructs; Table S2: List of all detected Beclin 1 interactions, ranked by SAINT probability.
