# Supplementary material for: ER-Targeted Beclin 1 Supports Autophagosome Biogenesis in the Absence of ULK1 and ULK2 Kinases
Source: Cells. 2019 May 17;8(5):475. doi: 10.3390/cells8050475 (PMC6562811; doi:10.3390/cells8050475)
Supplement: Supplementary file 1 [file cells-08-00475-s001.zip › Anwar-et-al-Supplements/Supplementary-Figures-Table1.pdf]

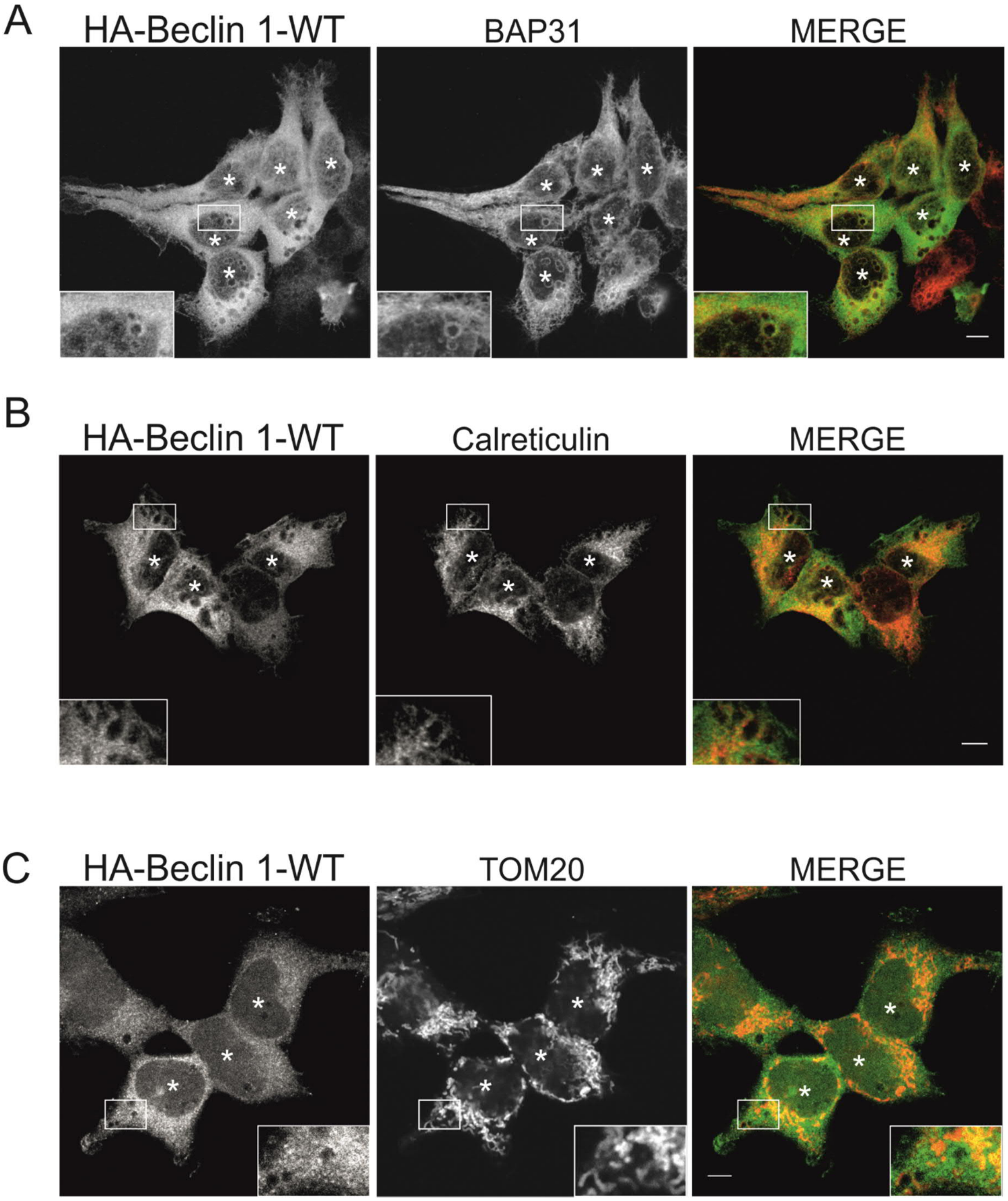

Figure S1: Subcellular localization of wild type Beclin 1 in HEK293 cells inducibly expressing Twin-StrepII-HA-tagged Beclin 1-WT. Expression was induced with 2 μg/ml tetracycline for 24 h. Cells were starved for 1 h and labelled with anti-HA (green) and anti-BAP31 (A) or anti-calreticulin (B) as ER markers, or anti-TOM20 as a mitochondrial marker (C). The ER and mitochondrial markers are shown in red in the merged panels. Images were taken with a confocal microscope and one optical section is shown. Cells expressing the wild type Beclin 1 construct are indicated by asterisks. Note that the overall staining patterns of Beclin 1 and the ER markers are similar to some extent, while those of Beclin 1 and the mitochondrial marker are different. Scale bars, 10 μm.

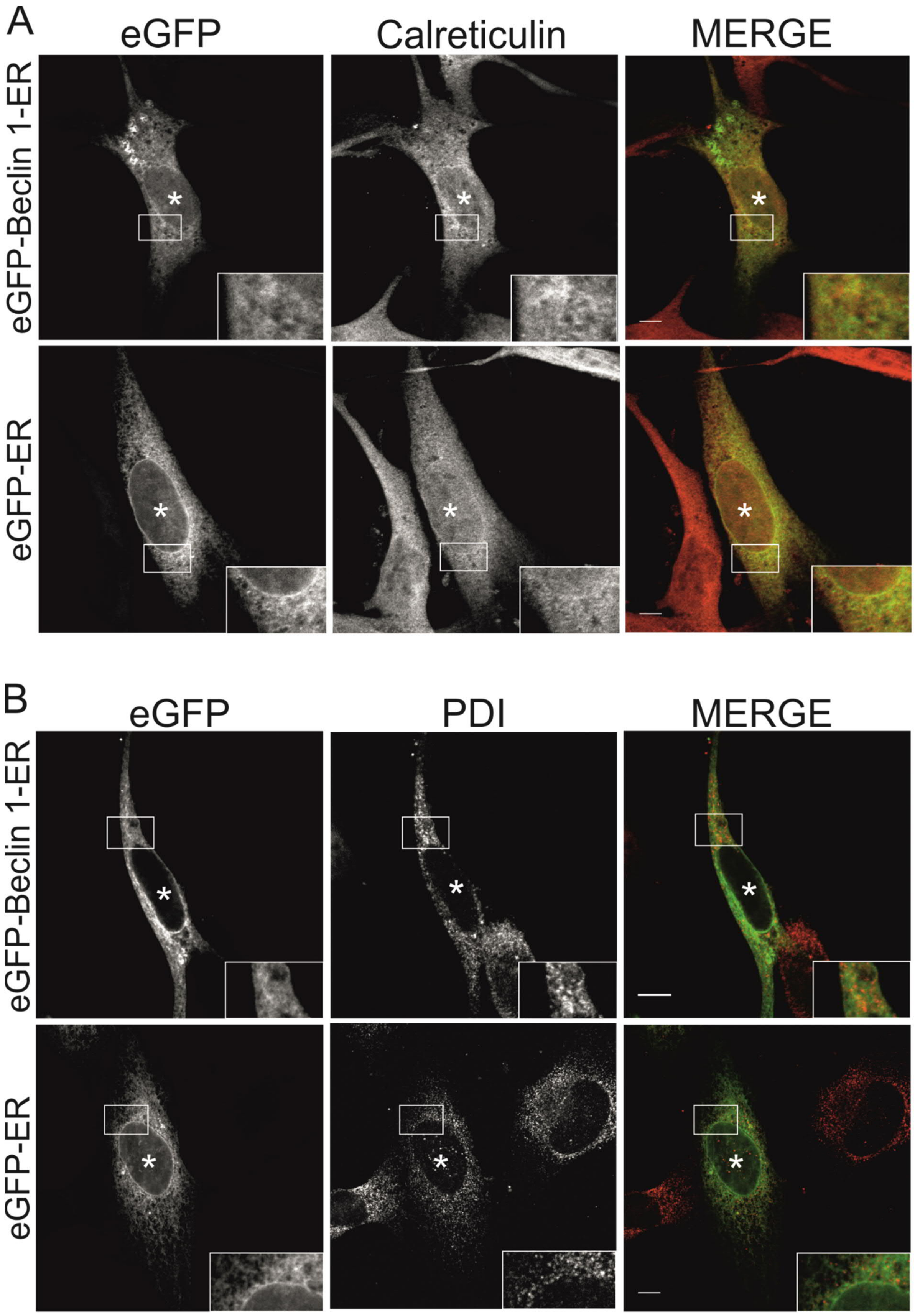
A
eGFP
Calreticulin
MERGE
eGFP-Beclin 1-ER
eGFP-ER
B
eGFP
PDI
MERGE
eGFP-Beclin 1-ER
eGFP-ER

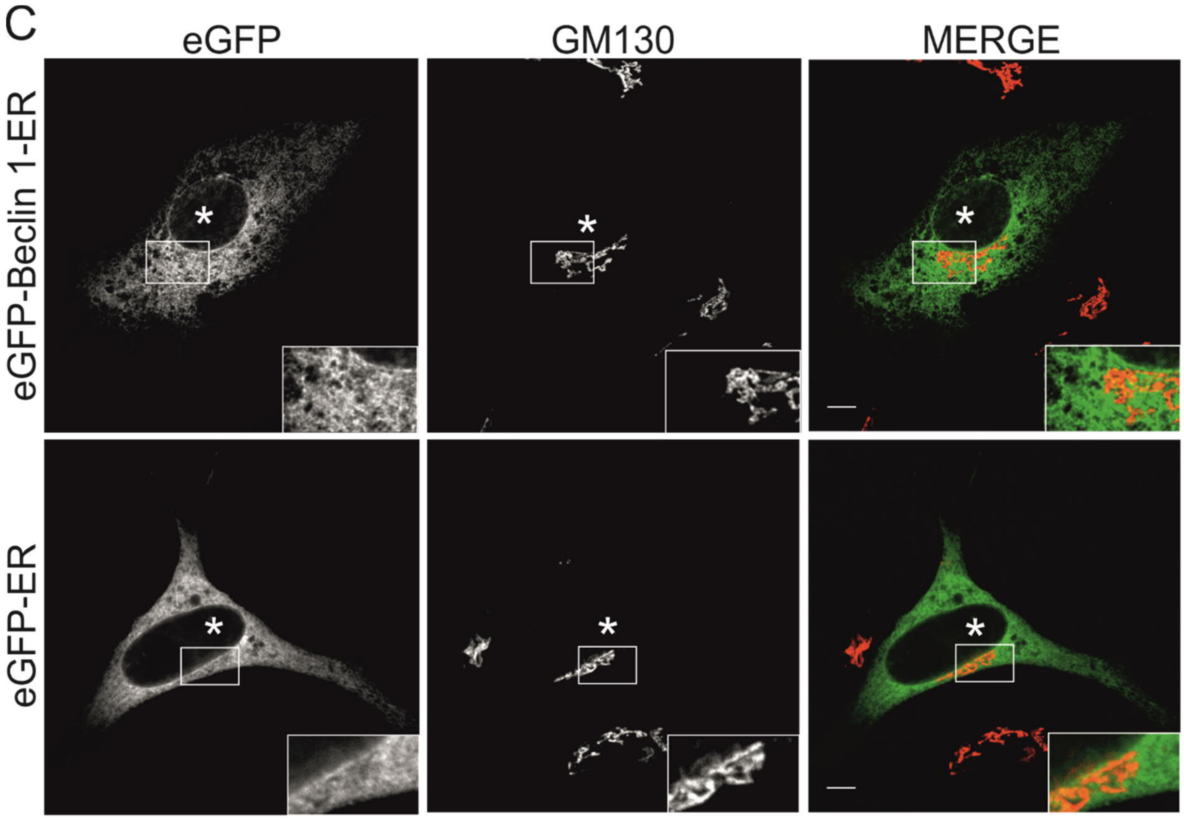

Figure S2: Colocalization of eGFP-Beclin 1-ER and eGFP-ER with ER markers calreticulin and protein disulphide isomerase (PDI), and Golgi marker GM130, in MEF-WT cells. The cells were transfected with eGFP-Beclin 1-ER or eGFP-ER , starved for 1 h and labelled against calreticulin (A), PDI (B), or GM130 (C). In the merged images, eGFP is green, and the ER and Golgi markers are red. Images were taken with a confocal microscope and one optical section is shown. Expressing cells are indicated by asterisks. Note that the overall staining patterns of Beclin 1-ER and the ER markers, especially calreticulin, are similar, while those of Beclin 1-ER and the Golgi marker are different. Scale bars, 10 μm.

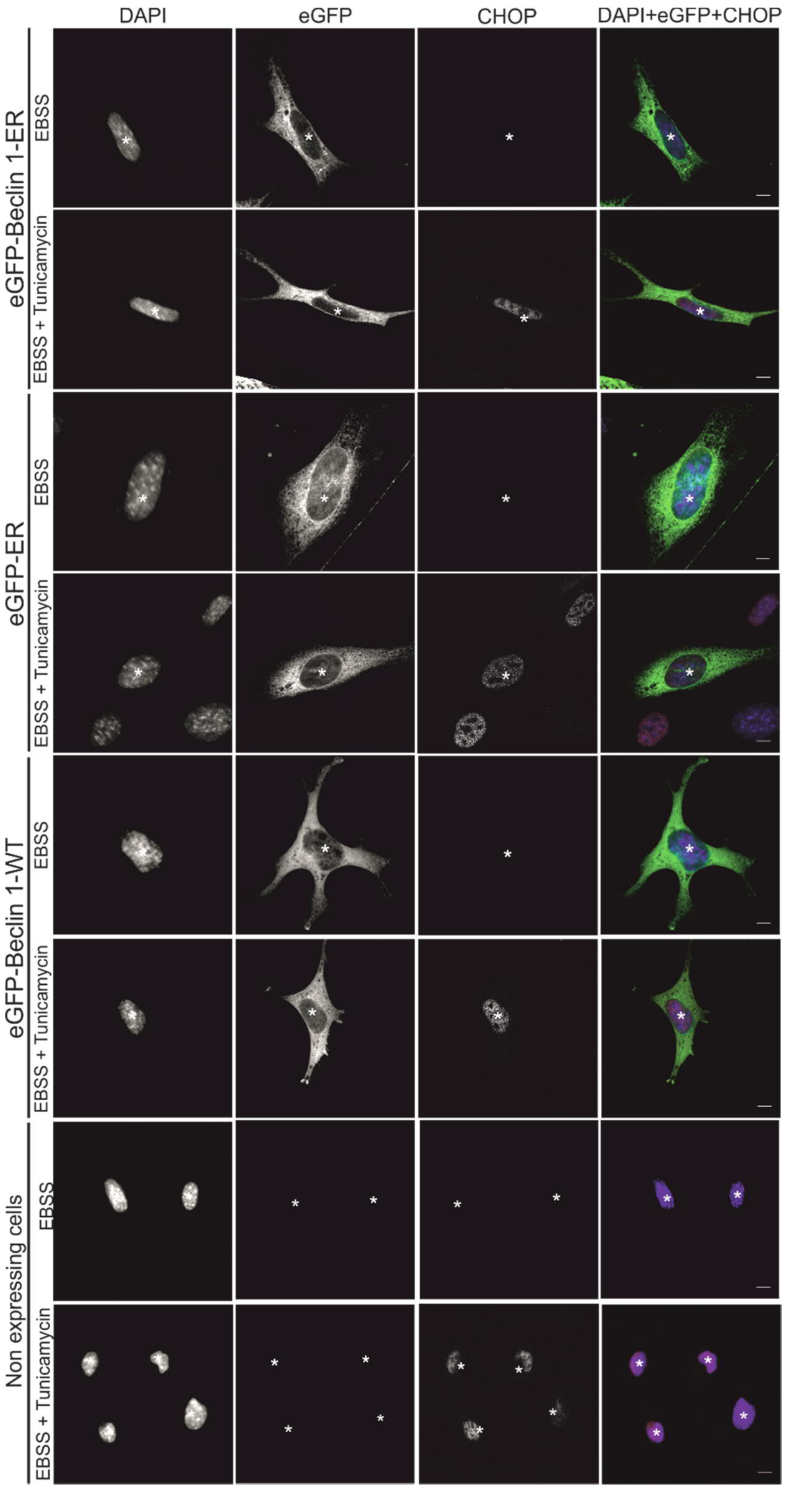

Figure S3: Expression of eGFP-Beclin 1-ER in MEF-WT cells does not induce ER stress. Appearance of the transcription factor CHOP in the nucleus was used to monitor ER stress, and tunicamycin treatment was used as a positive control. MEF-WT cells were transfected with eGFP-Beclin 1-ER, eGFP-ER or eGFP-Beclin 1-WT as indicated. Cells were starved for 1 h with or without 10 μg/ml tunicamycin and labelled with anti-CHOP. Asterisks indicate nuclei of cells expressing the eGFP-tagged constructs, or nuclei of non-expressing cells. Colors in the merged panels: DAPI blue, eGFP green, CHOP red. All images were taken with a confocal microscope and one optical section is shown. Scale bars, 10 μm.

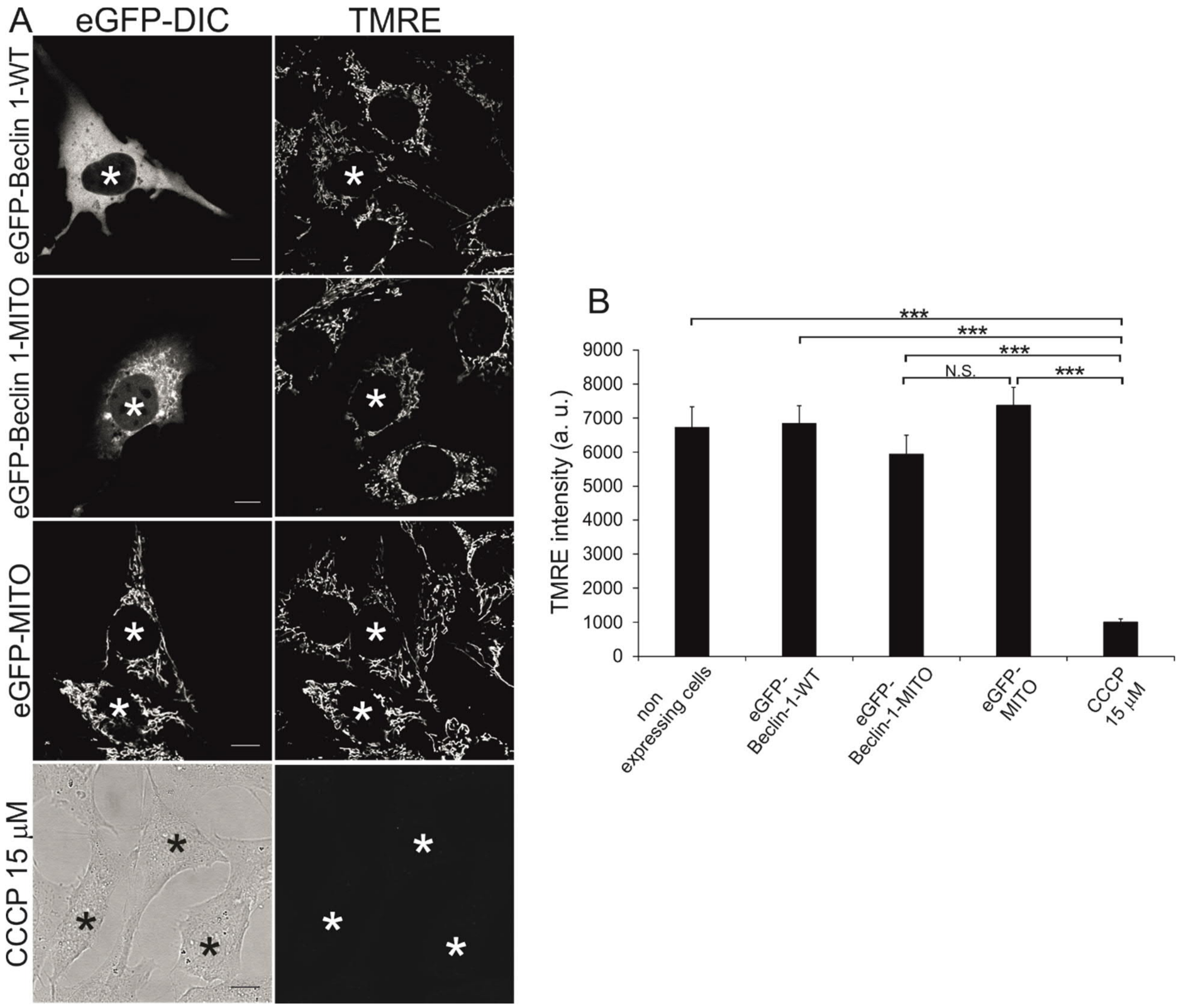

Figure S4: Expression of eGFP-Beclin 1-MITO in MEF-WT cells does not induce mitochondrial stress as detected using tetramethylrhodamine ethyl ester (TMRE) to monitor membrane potential. Carbonyl cyanide m-chlorophenyl hydrazone (CCCP) treatment was used as positive control to induce mitochondrial depolarization. (A) MEF-WT cells were transfected with eGFP-Beclin 1-WT, eGFP-Beclin 1-MITO, or eGFP-MITO, and starved for 1 h. TMRE (50 nM, 20 min) was used to monitor mitochondrial membrane potential in live cells without or with CCCP (15 μM, 20 min). Images were taken with a confocal microscope. Scale bars, 10 μm. (B) CellProfiler was used to quantify the TMRE intensity. Results are shown as mean and SEM of 20 cells (N=20). One-way ANOVA followed by Tukey Kramer post hoc test was used to test statistical significance. ***$p < 0.001$. N.S., not statistically significant ($p > 0.05$).

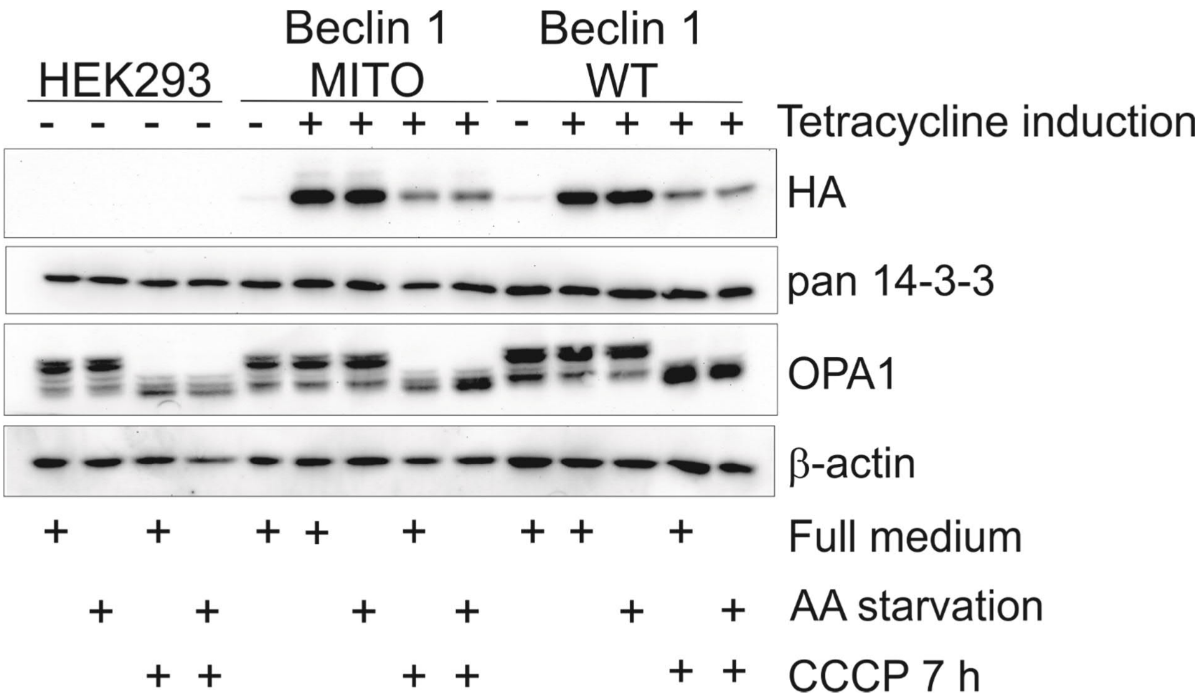

Figure S5: Expression of Twin-StrepII-HA-Beclin 1-MITO in HEK293 cells does not induce mitochondrial stress as detected by OPA1 cleavage. HEK293 cells inducibly expressing Twin-StrepII-HA-Beclin 1 constructs, Beclin 1-WT or Beclin 1-MITO, were induced by adding 2 μg/ml tetracycline to the culture medium for 24 h. Parental HEK293 cells were used as control. 30 μM CCCP was used as a positive control to depolarize mitochondrial membrane potential. Cells were kept in full medium or starved in amino-acid (AA) free medium for 1 h (without or with CCCP). Cell lysates were analysed by Western blotting with HA and OPA1 antibodies. Pan 14-3-3 and β-actin were used as loading controls. The experiment was repeated three times with similar results.

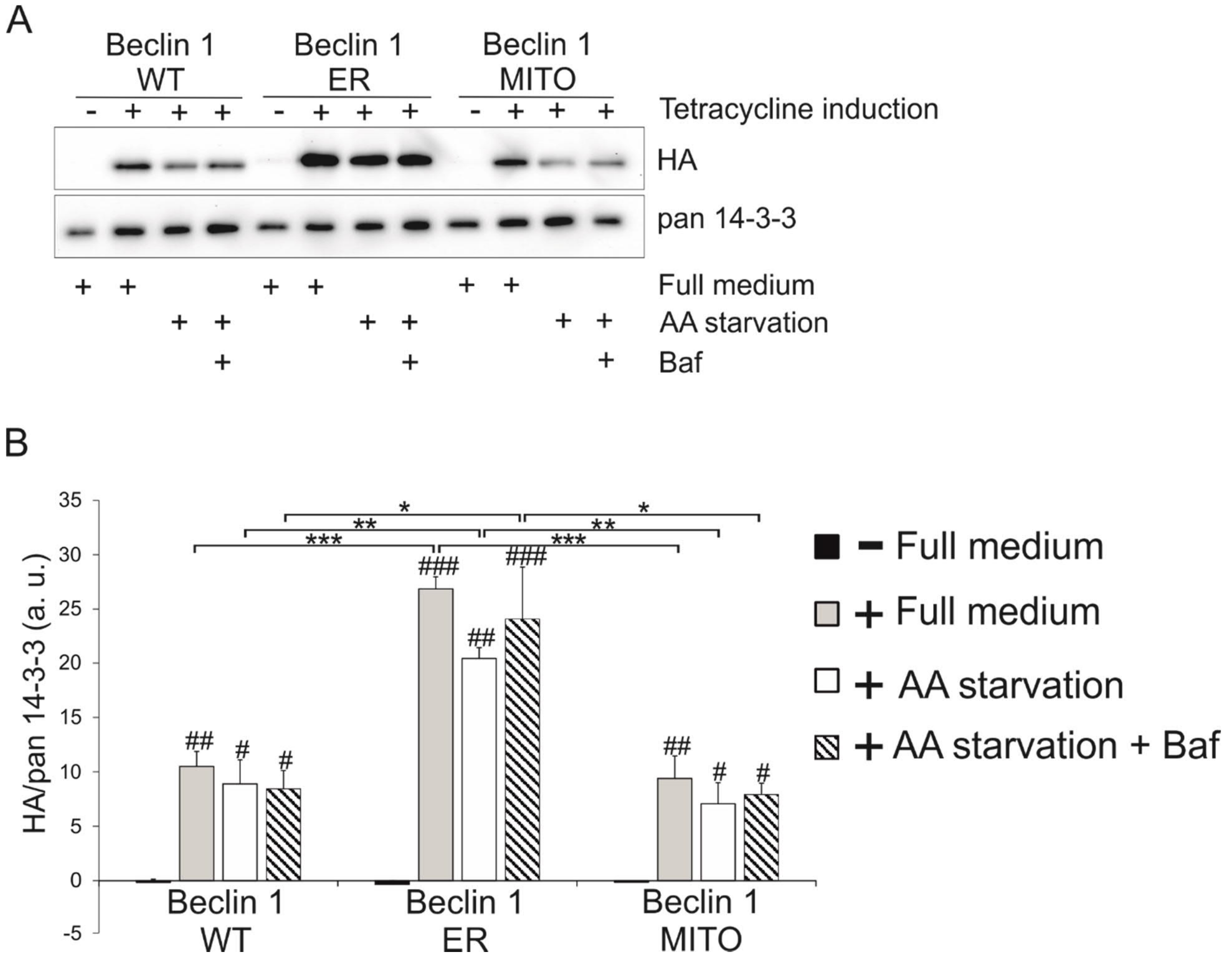

Figure S6: Expression levels of Twin-StrepII-HA-Beclin 1 constructs in HEK293 cells. HEK293 cells stably expressing the Beclin 1 constructs were induced by adding 2 μg/ml tetracycline to the culture medium for 24 h. Cells were kept in full medium or starved in amino-acid (AA) free medium with or without 100 nM bafilomycin A1 for 2 h. (A) Cell lysates were analysed by Western blotting with anti-HA. Pan 14-3-3 was used as loading control. (B) Quantification of the expression levels. The plus and minus indicate whether the expression was induced or not. Results are shown as mean and SEM of three biological replicas (N=3). One-way ANOVA followed by Tukey Kramer post hoc test was used to test statistical significance. *$p<0.05$, **$p<0.01$, ***$p<0.001$ for comparisons between the Beclin 1 stable cell lines as indicated. #$p<0.05$, ##$p<0.01$, ###$p<0.001$ for comparisons between the tetracycline induced samples and the non-induced sample within same cell line.

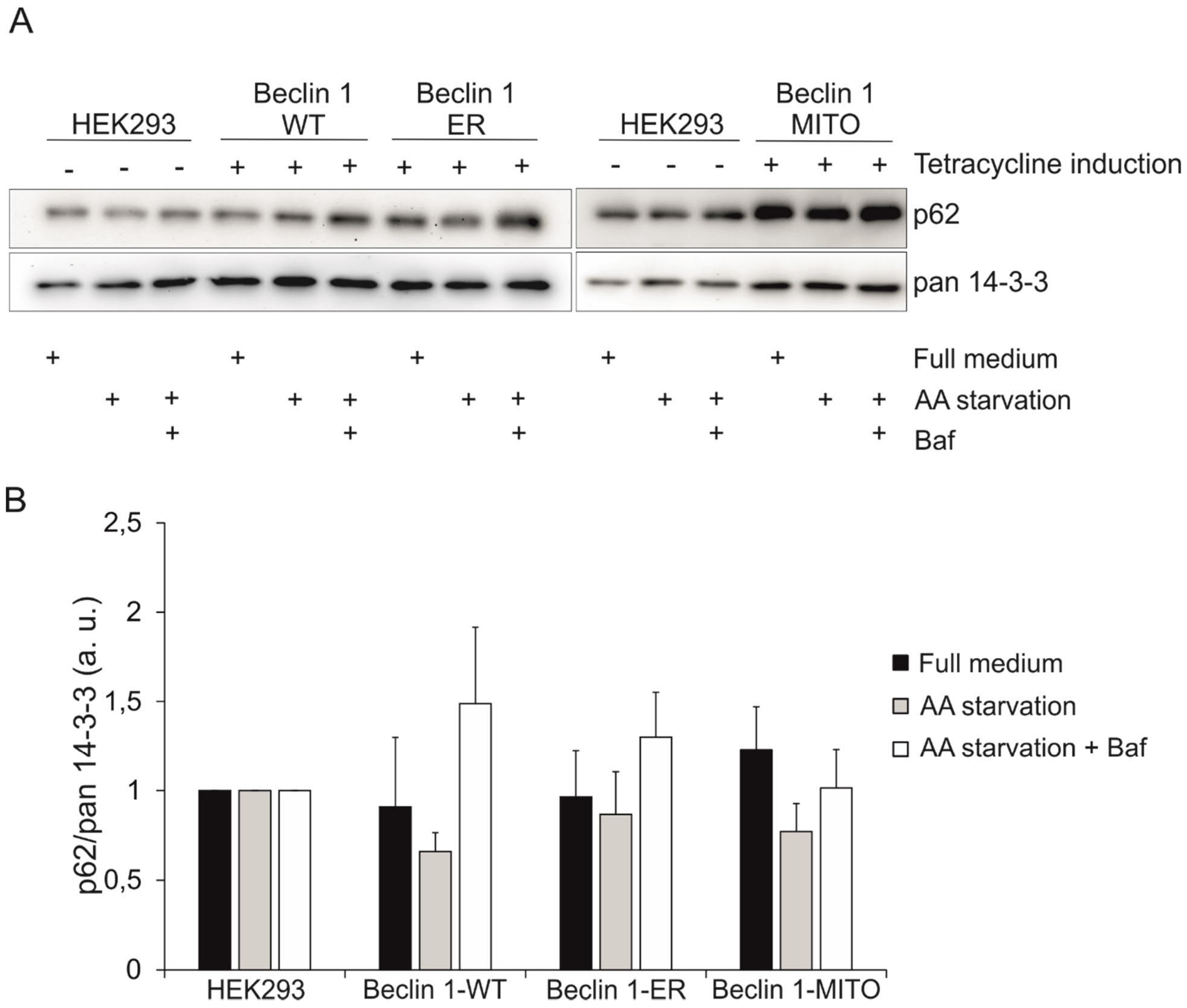

Figure S7: Effect of the Beclin 1 constructs on p62 levels in HEK293 cells stably expressing Twin-StrepII-HA-tagged Beclin 1. The cells were induced by adding 2 μg/ml tetracycline to the culture medium for 24 h. Normal HEK293 were used as control. Cells were kept in full medium or starved in amino-acid (AA) free medium with or without 100 nM bafilomycin A1 for 2 h. (A) Cell lysates were analysed by Western blotting with anti-p62. Pan 14-3-3 was used as loading control. Samples in the left and right boxes were run in different gels. (B) Relative p62 levels (p62/pan14-3-3) normalized to parental HEK293 cell line. Results are shown as mean and SEM of three biological replicas (N=3). One-way ANOVA followed by Tukey Kramer post hoc test, used to test statistical significance, showed that the differences were not significant.

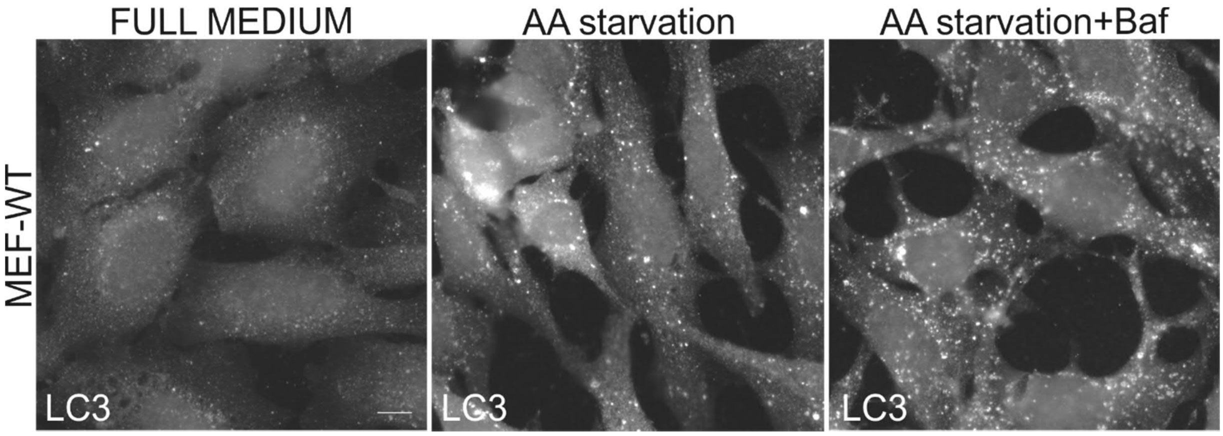

Figure S8: Representative immunofluorescence images for LC3 labelling in non-expressing MEF-WT. The cells were kept in full medium or starved in amino-acid-free medium with or without 100 nM bafilomycin A1 for 1 h, fixed and labelled with anti-LC3. Quantification is shown in Figure 6.

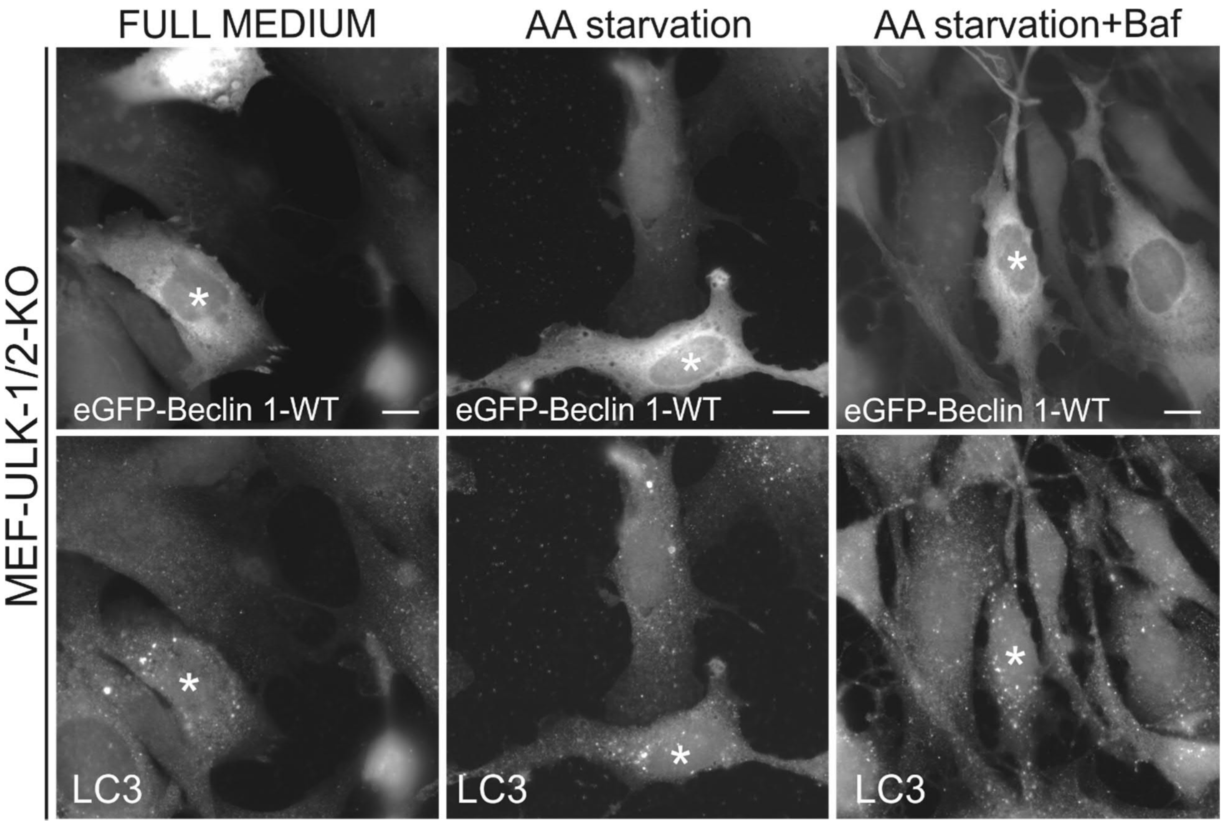

Figure S9: Representative immunofluorescence images for the LC3 labelling in MEF-ULK1/2-KO expressing eGFP-Beclin 1-WT. The cells were transfected with eGFP-Beclin 1-WT, kept in full medium or starved in amino-acid-free medium with or without 100 nM bafilomycin A1 for 1 h, fixed and labelled with anti-LC3. Asterisks indicate cells expressing eGFP-Beclin 1-WT. Scale bars, 10 μm. Quantification is shown in Figure 6.

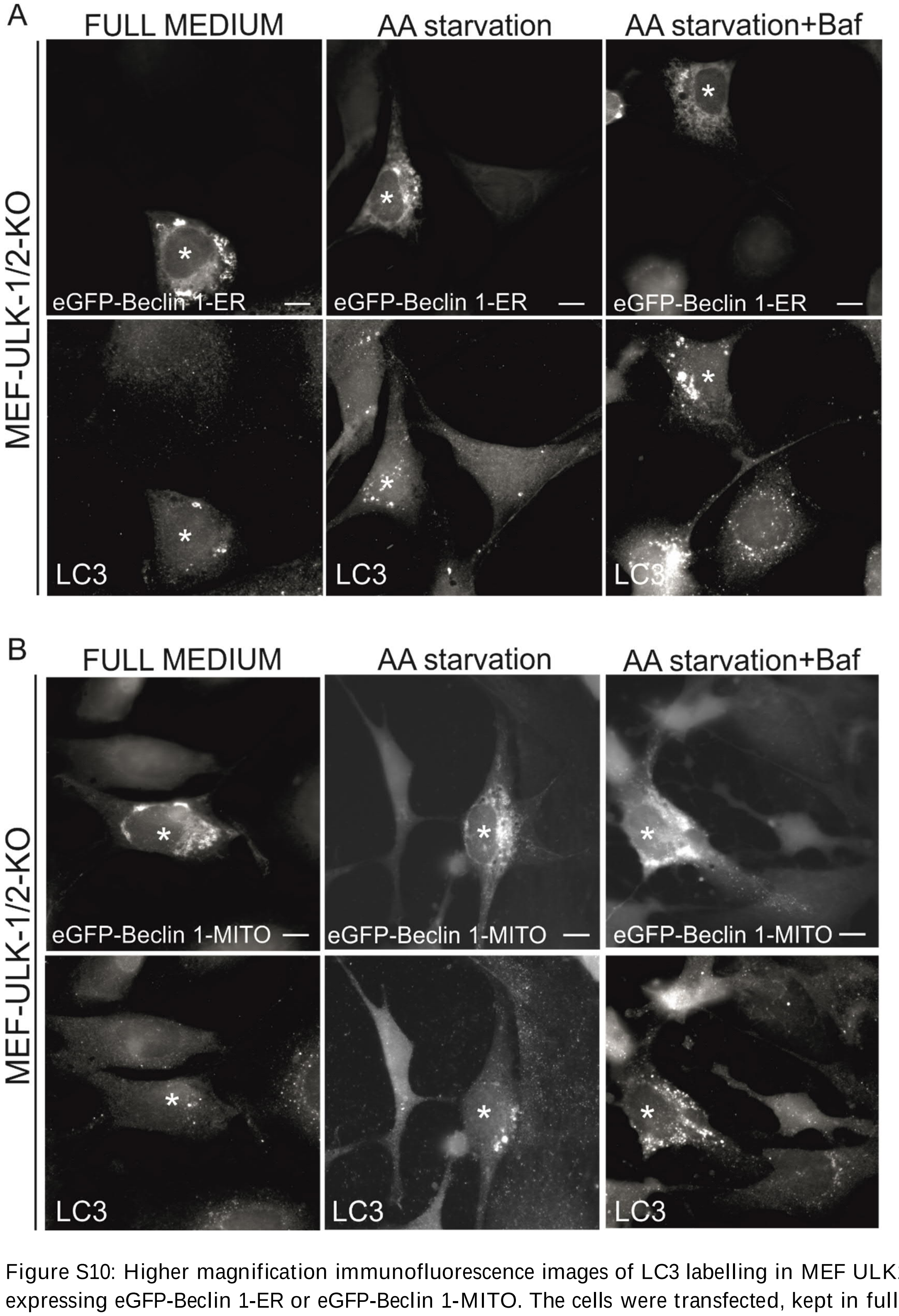

Figure S10: Higher magnification immunofluorescence images of LC3 labelling in MEF ULK1/2-KO cells expressing eGFP-Beclin 1-ER or eGFP-Beclin 1-MITO. The cells were transfected, kept in full medium or starved in amino-acid-free medium with or without 100 nM bafilomycin A1 for 1 h, fixed and labelled with anti-LC3. (A) eGFP-Beclin 1-ER (B) eGFP-Beclin 1-MITO. Asterisks indicate cells expressing the Beclin 1 constructs. Scale bars, 10 μm. Quantification is shown in Figure 6.

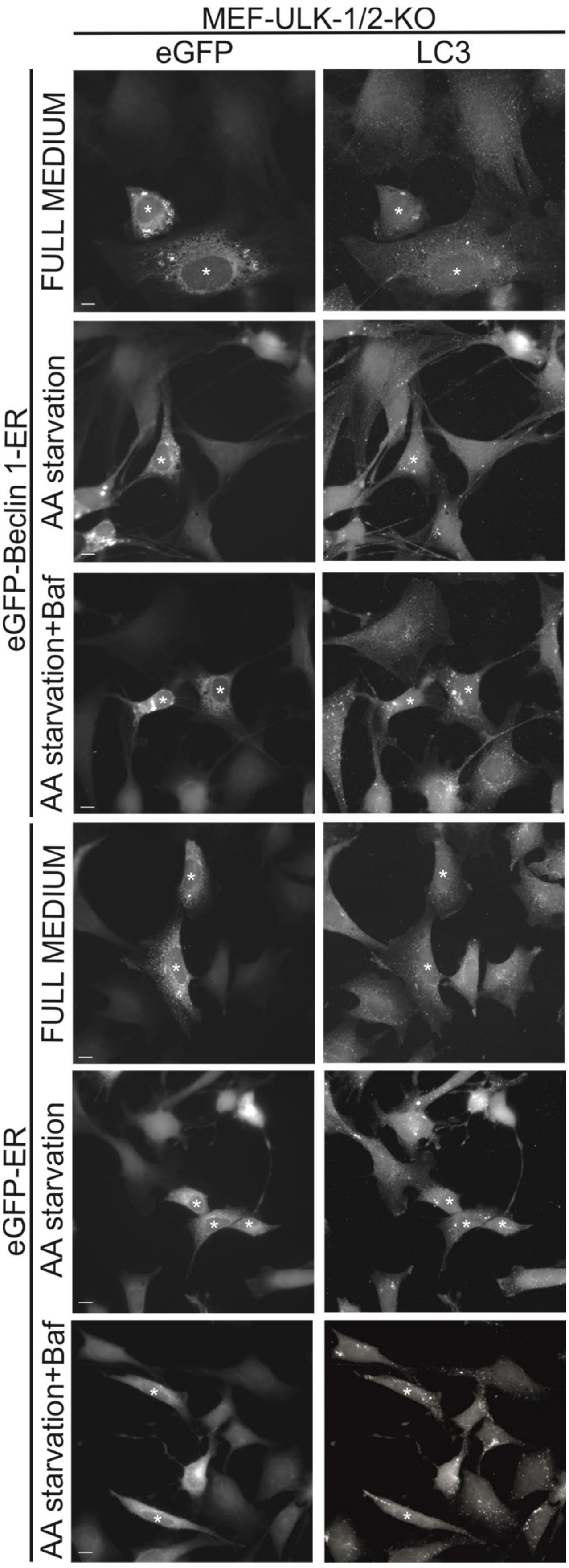

Figure S11: Representative immunofluorescence images for the quantification of vesicular LC3 in MEF-ULK1/2-KO cells expressing eGFP-Beclin 1-ER or eGFP-ER (related to Figure 6). The cells were transfected, kept in full medium or starved in amino-acid (AA) free medium with or without 100 nM bafilomycin A1 for 1 h, fixed and labelled with anti-LC3. Scale bars, 10 μm.

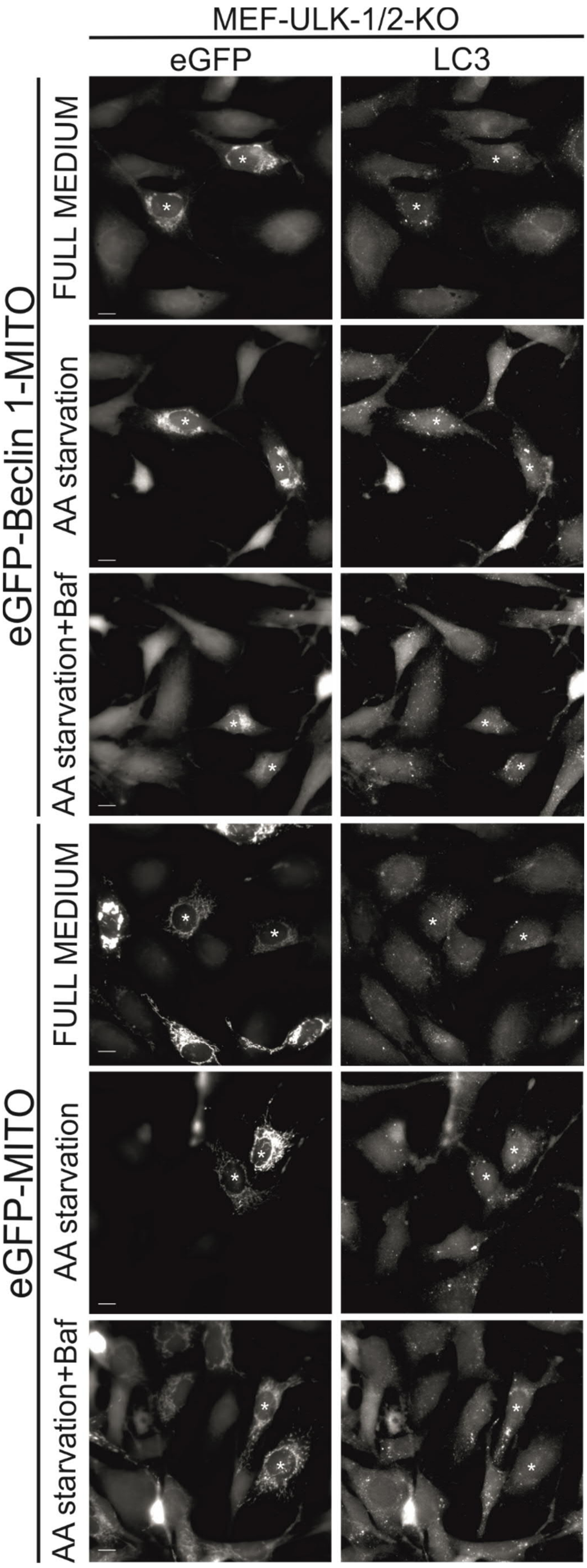

Figure S12: Representative immuno-fluorescence images for the quantification of vesicular LC3 in MEF-ULK1/2-KO cells expressing eGFP-Beclin 1-MITO or eGFP-MITO (related to Figure 6). The cells were transfected, kept in full medium or starved in amino-acid (AA) free medium with or without 100 nM bafilomycin for 1 h, fixed and labelled with anti-LC3. Scale bars, 10 μm.

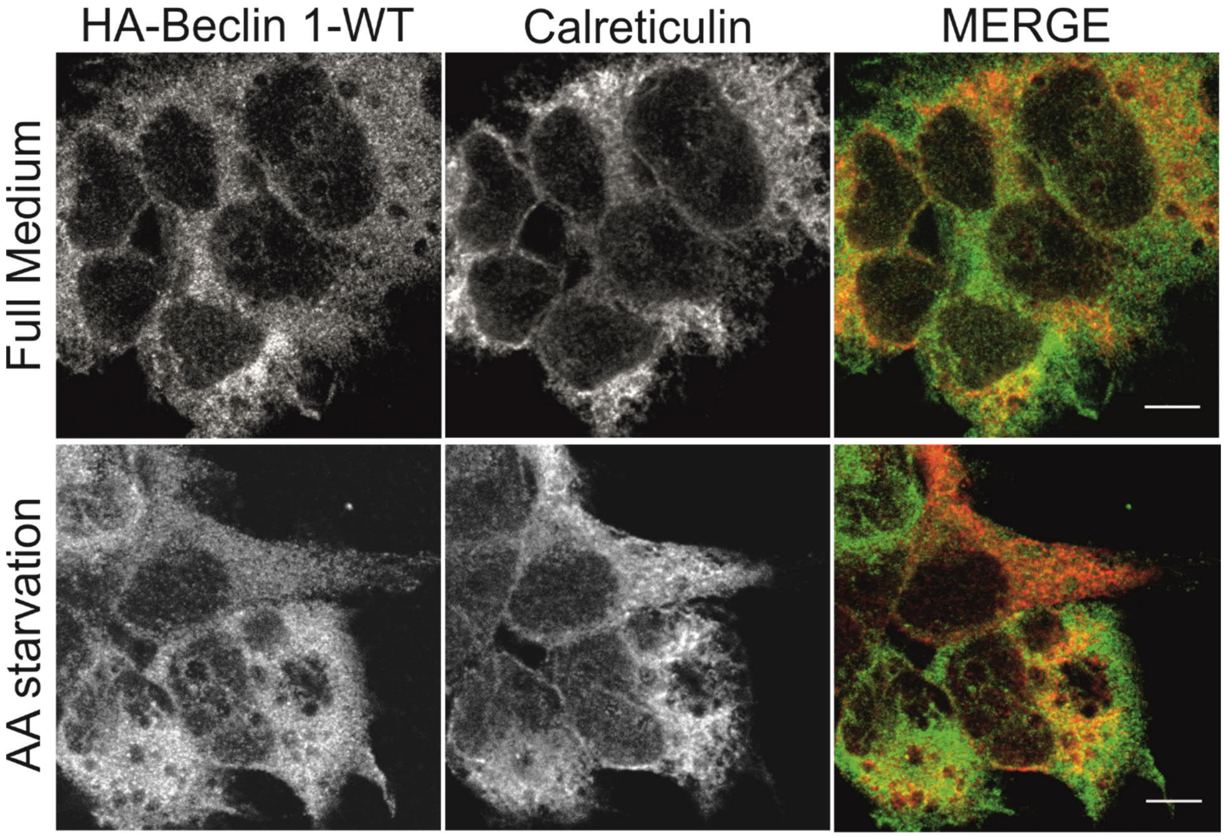

Figure S13: Immunofluorescene staining of Beclin 1-WT and the ER marker calreticulin in non-starved and amino-acid starved HEK293 cells inducibly expressing Twin-StrepII-HA-Beclin 1-WT. The cells were induced with 2 μg/ml tetracycline for 24 h, either kept in full medium or starved in amino-acid (AA) free medium for 1 h, fixed and labelled with anti-HA (green) and anti-calretculin (red). Images were taken with a confocal microscope. Scale bars, 10 μm. Quantification of colocalization is shown in Figure 9A.

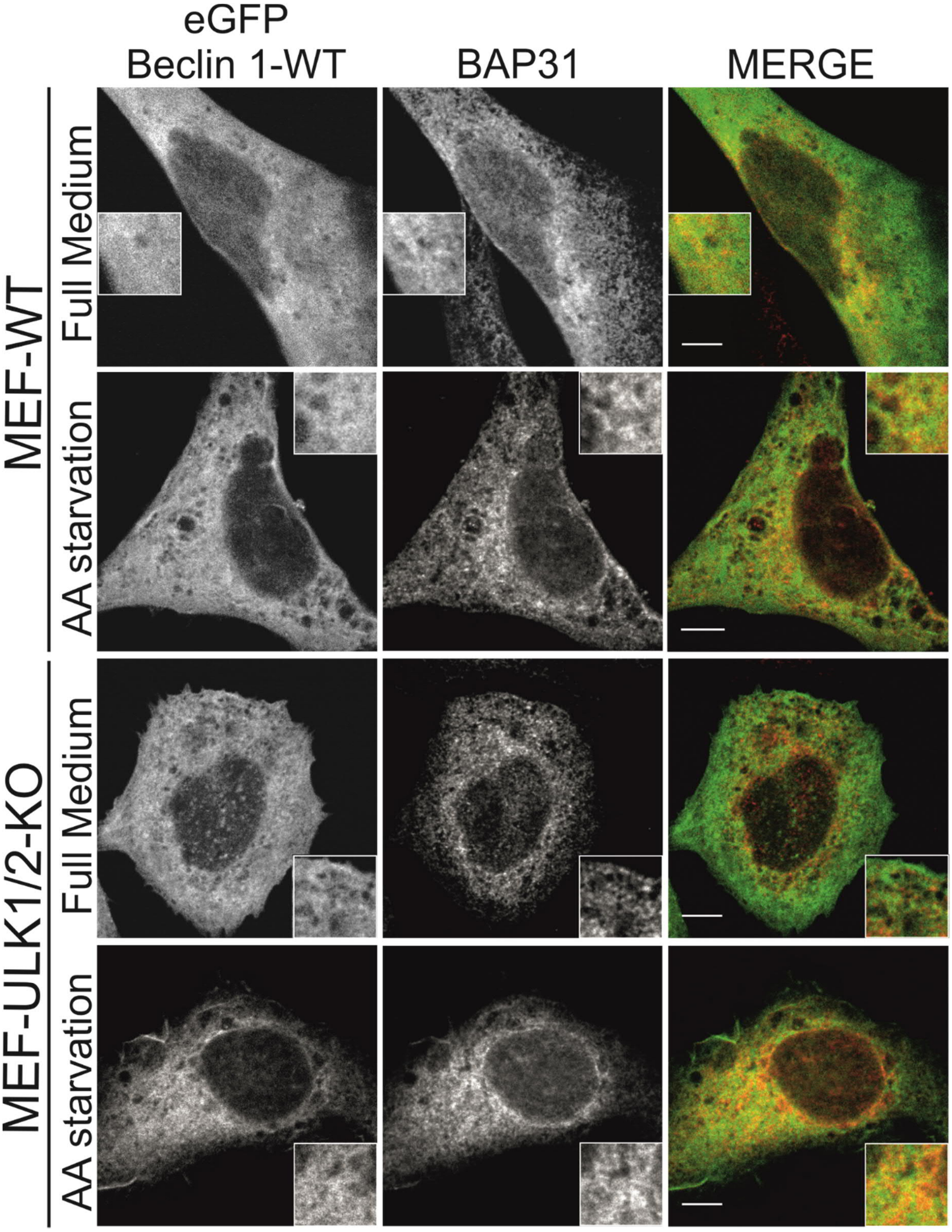

Figure S14. Colocalization of eGFP-Beclin 1-WT with the ER marker BAP31 in non-starved and amino-acid starved MEF cells. MEF-WT and MEF-ULK1/2-KO cells transiently expressing eGFP-Beclin 1-WT were either kept in full medium or starved in amino-acid (AA) free medium for 1 h, fixed and labelled anti-BAP31 (red). Images were taken with a confocal microscope. Scale bars, 10 μm. Quantification of colocalization is shown in Figure 9B.

SEQUENCE FUNCTION	PRIMER SEQUENCES
Mitochondrial targeting for cloning into pCDNA3.1(+) FLAG-Beclin 1 vector (Acta)	BclACTA-F1 5´gcttgggtgtcctcacaattttataacaaattaattcttgcaatgttagct3´<br>BclACTA-F2 5´attggcgtgttctctttaggggcgtttatcaaaattattcaattaagaaaaa3´<br>BclACTA-R1 5´gcggccgcttaattatttttcttaattgaataattttgataaacgcccct3´<br>BclACTA-R2<br>5´ctaaagagaacacgccaatagctaacattgcaagaattaatttgttataaaattgtg3´
Endoplasmic reticulum targeting) for cloning into pCDNA3.1(+) FLAG-Beclin 1 vector (Cytochrome b5)	Bclb5-F1 5´gcttgggtgtcctcacaattttataacaaaatcactactattgattctagttcc3´<br>Bclb5-F2 5´gaccaactgggtgatccctgccatctctgcagtggccgtcgccttgatgtatcgc3´<br>Bclb5-R1 5´gcggccgctcagtcctctgccatgtataggcgatacatcaaggcgacggcc3´<br>Bclb5-R2<br>5´cagggatcacccagttggtccaccaactggaactagaatcaatagtagtgattttg3´
Flanking sequences for cloning into pCDNA3.1(+) FLAG-Beclin 1 vector	Bcl-F 5´gcttgggtgtcctcacaat3´ (used for both the Beclin 1-Acta and Beclin 1- Cytochrome b5 cloning)<br>Cytb5-R 5´gcggccgctcagtcctctg3´ (used for Beclin 1- Cytochrome b5)<br>ACTA-R 5´gcggccgcttaattatttttct3´ (used for Beclin 1-Acta)
Flanking sequences for cloning into pEGFP-C1 vector	F1 lnk Becl 5´ggcgggtctggcatggaagggtctaag3´<br>F2 lnk lnk Becl 5´tctagtggcagtggcgggtctggcatg3´<br>F3 BamH lnk Becl ggatccggcggctctagtggcagtggc3´<br>R-Beclin 5´cgagaagctttcatttgttataaaattgtgagg3´<br>cytb5 R 5´gcggccgctcagtcctctg3´<br>ACTA R 5´gcggccgcttaattatttttct3´
Mitochondrial targeting control (Acta)	F 5'aatggatccttaattcttgcaatgttagc3'<br>R 5'ttatctagattaattatttttcttaattgaataa3'
Endoplasmic reticulum targeting control (Cytochrome b5)	F1 5´cgggtctggcatcactactattgattctagt3´<br>F2 5´cagtggcgggtctggcatcact3´<br>F3 5´tctagtggcagtggcgggtct3´<br>F4 5´ggcggctctagtggcagtg3´<br>F5 5´tttagatctggcggctctagtgg3´<br>R 5´tataagctttcagtcctctgccatgtatag3´
Gateway 1st PCR reaction	Beclin-F 5´aaaaagcaggctccaccatggtgagcaagggcga3´ (used for all Beclin 1 constructs)<br>WT-R 5´agaaagctgggtctcatttgttataaaattgtgaggacac3´<br>Acta-R 5´agaaagctgggtcgcggccgcttaattatttttt3´<br>CytoCb5-R 5´agaaagctgggtcgctcagtcctctgccatgta3´
Gateway 2nd PCR reaction	attB1 5´ggggacaagtttgtacaaaaaagcaggct3'<br>attB2 5'ggggaccactttgtacaagaaagctgggt3'

Table 1: List of oligonucleotide primers used for the construction of the Beclin 1 constructs.
